# Gridded maps of wetlands dynamics over mid-low latitudes for 1980–2020 based on TOPMODEL

**DOI:** 10.1038/s41597-022-01460-w

**Published:** 2022-06-18

**Authors:** Yi Xi, Shushi Peng, Agnès Ducharne, Philippe Ciais, Thomas Gumbricht, Carlos Jimenez, Benjamin Poulter, Catherine Prigent, Chunjing Qiu, Marielle Saunois, Zhen Zhang

**Affiliations:** 1 Sino-French Institute for Earth System Science, Laboratory for Earth Surface Processes, College of Urban and Environmental Sciences, and, Peking University, Beijing, 100871 China; 2 Sorbonne Université, CNRS, EPHE, Laboratoire METIS (Milieux environnementaux, transferts et interaction dans les hydrosystèmes et les sols), 75005 Paris, France; 3 Laboratoire des Sciences du Climat et de l’Environnement, LSCE/IPSL, CEA-CNRS-UVSQ, Université Paris-Saclay, 91191 Gif-sur-Yvette, France; 4 Center for International Forestry Research (CIFOR), Bogor, Indonesia; 5 Karttur AB, Stockholm, Sweden; 6 Estellus, Paris, France; 7 Sorbonne Université, Observatoire de Paris, Université PSL, CNRS, LERMA, Paris, France; 8 NASA Goddard Space Flight Center, Biospheric Science Laboratory, Greenbelt, MD 20771 USA; 9 Department of Geographical Sciences, University of Maryland, College Park, USA

**Keywords:** Hydrology, Freshwater ecology, Limnology

## Abstract

Dynamics of global wetlands are closely linked to biodiversity conservation, hydrology, and greenhouse gas emissions. However, long-term time series of global wetland products are still lacking. Using a diagnostic model based on the TOPography-based hydrological MODEL (TOPMODEL), this study produced an ensemble of 28 gridded maps of monthly global/regional wetland extents (with more reliable estimates at mid-low latitudes) for 1980–2020 at 0.25° × 0.25° spatial resolution, calibrated with a combination of four observation-based wetland data and seven gridded soil moisture reanalysis datasets. The gridded dynamic maps of wetlands capture the spatial distributions, seasonal cycles, and interannual variabilities of observed wetland extent well, and also show a good agreement with independent satellite-based terrestrial water storage estimates over wetland areas. The long temporal coverage extending beyond the era of satellite datasets, the global coverage, and the opportunity to provide real-time updates from ongoing soil moisture data make these products helpful for various applications such as analyzing the wetland-related methane emission.

## Abstract (summary)

**Table:** 

Measurement(s)	wetland area
Technology Type(s)	computational modeling technique
Factor Type(s)	geographic location • temporal interval
Sample Characteristic - Environment	land
Sample Characteristic - Location	global

## Background & Summary

Wetlands are usually defined as ecosystems where saturation or inundation dominate the soil development and determines plant species composition^1^, including shallow inland water bodies, peatlands, mineral wetlands, seasonal and permanent floodplains and so on. In addition to supporting plant and animal species to maintain regional biological diversity, wetlands are very important for regional water cycles and water quality by regulating river flows and groundwater recharge and removing nutrients responsible for eutrophication^2,3^. Moreover, pristine natural wetlands represent large carbon stocks and permanent sinks of carbon dioxide (CO_2_) and methane (CH_4_) sources. In recent studies of the Global Methane Budget covering the period 2000–2017, natural wetlands are estimated to be the largest natural CH_4_ source, emitting 102–182 Tg CH_4_ yr^−1^ (27–49% of all natural sources) using bottom-up models and 155–200 Tg CH_4_ yr^−1^ (72–93%) using atmospheric inversions from 2008 to 2017 (ref. ^4^).

To understand the role of wetlands on global hydrological and biogeochemical cycles, an accurate estimation of wetland extent and its temporal variations is required^4,5^. Global wetland inventories are usually static and only document long-term (usually maximum) wetland extent, thereby lacking information on temporal dynamics^6–9^. Besides, given the lack of accurate definition, the small spatial scale of many wetland systems, and inventory sampling limitations, estimates of global wetland extent have considerable discrepancies with a range of 2.1–29.8 Mkm^2^ (ref. ^10^). Satellite-based datasets from the visible, infrared, or microwave bands have been developed to characterize the spatiotemporal dynamics of wetlands^11,12^, but with different degrees of success depending on the environments (Table 1). The optical satellite data such as the global surface water from the European Commission’s Joint Research Centre (hereafter JRC)^13^ provides a high spatial resolution (30 m) but only detects open water bodies without dense vegetation for 1984–2015. Microwaves, including active and passive can penetrate dense vegetation and clouds but are limited by small spatial coverage or low spatial resolution, respectively^12^. Multi-sensors datasets such as the Global Inundation Extent from Multi-satellites version 2 (GIEMS-2)^14^ and the Surface WAter Microwave Product Series (SWAMPS)^15^ capitalize the strengths of different satellites and provide maximum information of surface inundated extent since the 1990s, but miss some small wetlands due to their coarse global 0.25° × 0.25° resolution^14^. Hence, mapping the long-term dynamics of wetland extent is still a challenge.

**Table 1:** Summary of global/regional wetland-related datasets from the literature. The four data sets used here to calibrate our model are in bold.

Name and reference	Period	Temporal resolution	Spatial resolution	Water body classes	Wetland area (Mkm^2^)
GLWD-3 (Lehner and Döll, 2004)	Static	—	30 arcsec (~1 km)	12 water classes (3 permanent water classes and 9 natural wetland classes)	10.9–12.8^a^
GIEMS-1 (Prigent *et al*.^34^)	1993–2007	Monthly	0.25° (~25 km)	Natural and artificial inundated areas (excluding larger lakes)	2.1–5.9^b^
ESACCI land cover (Herold *et al*.^36^)	1992–2018	Yearly	10 arcsec (~300 m)	Open water bodies	6.1
GIEMS-D15 (Fluet-Chouinard *et al*.^37^)	Static	—	15 arcsec (~500 m)	Inundated areas of global land surface	6.5–12.1^b^
JRC (Pekel *et al*.^13^)	1984–2018	Monthly	~30 m	Surface water	4.5^c^
**G2017 (Gumbricht** ***et al***.^9^)	Static	—	~232 m	Tropical and subtropical (**40°N–60°S**) wetland and peatland areas	4.7
**RFW (Tootchi** *et al*.^10^)	Static	—	15 arcsec (~500 m)	Regular flooded wetlands (excluding large permanent lakes)	12.9
SWAMPS (Jensen *et al*.^15^)	1992–2018	Monthly	0.25° (~25 km)	All terrestrial water fractions	3.6–6.2^b^
**GIEMS-2 (Prigent** ***et al.***^14^)	1992–2015	Monthly	0.25° (~25 km)	Natural and artificial inundated areas (excluding large lakes)	1.6–4.6^b^
BAWLD (Olefeldt *et al*.^45^)	Static	—	0.5° (~50 km)	Five wetland types including bog, fen, marsh, tundra wetland, and permafrost bog in the northern boreal and tundra biomes (>55°N)	2.8–3.8
**WAD2M (Zhang** ***et al***.^33^)	2000–2018	Monthly	0.25° (~25 km)	Surface inundation areas, including wetlands from under dense canopies, excluding lakes, rivers, ponds, and rice agriculture	3.1–5.6^b^

^a^The range is determined according to the uncertain wetland fraction for three wetland classes in GLWD-3.

^b^The range indicates mean annual minimum and mean annual maximum wetland extent.

^c^The range indicates long-term maximum global surface water extent.

For modeling wetland areas, the simple hydrological model TOPMODEL (TOPography-based hydrological MODEL) developed 40 years ago^16^ has been widely used with different settings to delineate wetlands^16–20^. Owing to topography being the main factor determining water pathways, TOPMODEL uses a compound topographic index (CTI) to redistribute soil water in sub-grid elements of a larger land surface grid-cell or a catchment^21^. The always saturated or regularly saturated sub-grids can be regarded as wetlands. Global-scale CTI datasets usually have a high spatial resolution, for example, 30 arcsec for the HYDRO1k dataset developed by the U.S. Geological Survey and 15 arcsec for the CTI dataset of Marthews *et al*. (ref. ^22^) which results in high computational costs to diagnose wetland dynamics from the distributions of CTI in each grid cell. To improve this, TOPMODEL was developed to link the wetland fraction to the mean water table depth (WTD) (typically available at 0.25°–1° from global land surface models)^19,20,23,24^. For example, Niu *et al*. (ref. ^23^) developed a parameterization to treat subsurface runoff in TOPMODEL as a product of an exponential function of the mean WTD at 1° × 1° resolution. Stocker *et al*. (ref. ^20^) used an asymmetric sigmoid function to fit the relationship between the inundated fraction and mean WTD at 1° × 1° resolution. Such diagnostic algorithms make the global implementation of TOPMODEL to simulate wetland dynamics more tractable.

Following the TOPMODEL-based diagnostic model from Stocker *et al*. (ref. ^20^) and Xi *et al*. (ref. ^25^), this study produced an ensemble of 28 sets of monthly global/regional wetland maps for 1980–2020 at 0.25° × 0.25° spatial resolution based on seven reanalysis soil moisture (SM) datasets and four observation-based wetland data. Different SM datasets provide the uncertainties of inputs of the model while different wetland data cover different definitions of wetlands for the convenience of different research purposes. Evaluation against the corresponding wetland calibration data, independent wetland datasets, and satellite-based terrestrial water storage estimates suggests that the well-calibrated diagnostic model can capture the spatial distributions, seasonal cycles, and interannual variabilities of observed wetland extent well. Our intent is for this dataset to provide opportunities for long-term wetland-related studies beyond the era of satellite-based datasets.

## Methods

**Fig. 1:**
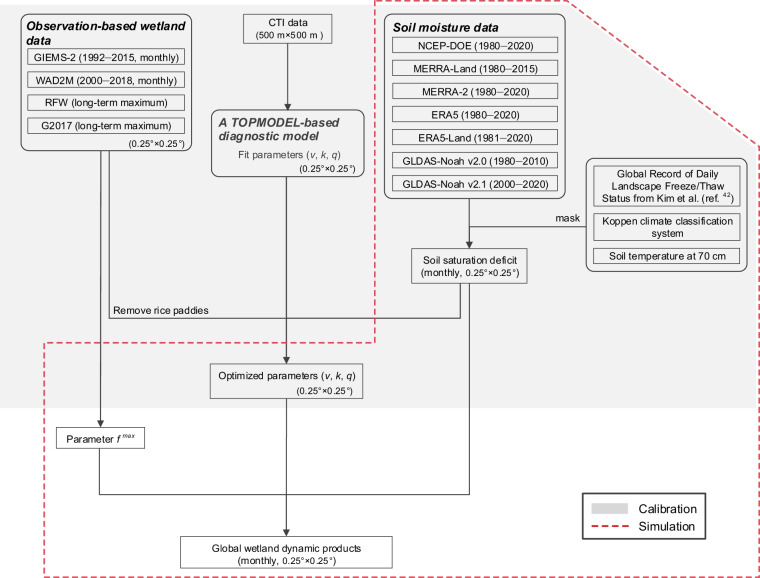
Diagram of workflow for parameter calibration and the simulation of global wetland dynamics.

The conceptual flow chart of the process is provided in Fig. 1. We used seven reanalysis SM data (Table 2) masked with soil temperature (ST) and soil freeze/thaw status to calculate water table depth, i.e. the input of TOPMODEL, given the obvious disagreements between the input datasets. The diagnostic algorithms based on TOPMODEL were used following Stocker *et al*. (ref. ^20^) and Xi *et al*. (ref. ^25^), where the optimized parameters were calibrated with long-term maximum wetland areas from four observation-based wetland datasets (Table 1). Details about these datasets and computational processing are shown as follows.

**Table 2:** Key characteristics of seven global soil moisture reanalysis data used in this study.

Dataset	Period (month)	Spatial resolution	Temporal resolution	Soil thickness	Data Access
NCEP-DOE	198001–202012	~210 km	Daily	0–0.1 m, 0.1–2 m	https://www.esrl.noaa.gov/psd/data/gridded/data.ncep.reanalysis2.gaussian.html
MERRA-Land	198001–201512	0.5° × 0.667°	Monthly	1.0–8.0 m	https://goldsmr2.gesdisc.eosdis.nasa.gov/data/MERRA_MONTHLY/MSTMNXMLD.5.2.0/
MERRA-2	198001–202012	0.5° × 0.625°	Monthly	1.3–8.5 m	https://goldsmr4.gesdisc.eosdis.nasa.gov/data/MERRA2_MONTHLY/M2TMNXLND.5.12.4/
ERA5	198001–202012	0.25° × 0.25°	Monthly	0–0.07 m, 0.07–0.28 m, 0.28–1 m, 1–2.89 m	https://cds.climate.copernicus.eu/cdsapp#!/dataset/reanalysis-era5-single-levels-monthly-means?tab = form
ERA5-Land	198101–202011	0.1° × 0.1°	Monthly	0–0.07 m, 0.07–0.28 m, 0.28–1 m, 1–2.89 m	https://cds.climate.copernicus.eu/cdsapp#!/dataset/reanalysis-era5-land-monthly-means?tab = form
GLDAS-Noah v2.0	198001–201412	0.25° × 0.25°	Monthly	0–0.1 m, 0.1–0.4 m, 0.4–1 m, 1–2 m	https://disc.sci.gsfc.nasa.gov/datasets/GLDAS_NOAH025_M_V2.0/summary?keywords = GLDAS
GLDAS-Noah v2.1	200001–202009	0.25° × 0.25°	Monthly	0–0.1 m, 0.1–0.4 m, 0.4–1 m, 1–2 m	https://disc.sci.gsfc.nasa.gov/datasets/GLDAS_NOAH025_M_V2.1/summary?keywords = GLDAS

### Reanalysis soil moisture datasets

Seven long-term reanalysis SM datasets used in this study include NCEP-DOE (National Centers for Environmental Prediction-the Department of Energy)^26^, MERRA-Land (the Modern-Era Retrospective Analysis for Research and Applications)^27^, MERRA-2 (ref. ^28^), GLDAS-Noah v2.0 (the Global Land Data Assimilation System)^29^, GLDAS-Noah v2.1 (ref. ^29^), ERA5 (European Environment Agency)^30,31^, and ERA5-Land^30,31^. Key characteristics of the seven SM data are listed in Table 2. The datasets differ by their spatial and temporal resolutions, the time-period they cover, as well as the definition of the soil layers. More details are provided for each dataset below.

- NCEP-DOE
  NCEP-DOE is an updated version of the National Centers for Environmental Prediction/National Center for Atmospheric Research (NCEP/NCAR) Reanalysis 1 project, which uses a state-of-the-art analysis/forecast system to perform data assimilation with past data from 1948 to the present^32^. NCEP-DOE features the newer physics and observed SM forcing and also eliminates several previous errors, such as oceanic albedo and snowmelt term during the entire period, and snow cover analysis error from 1974 to 1994 (ref. ^26^). With a spatial resolution of about 210 km, there are two vertical soil layers in NCEP-DOE for both SM and ST: 0–0.1 and 0.1–2 m.
- MERRA-Land and MERRA-2
  MERRA-Land soil moisture is generated by driving the Goddard Earth Observing System model version 5.7.2 (GEOS-5.7.2) with meteorological forcing from the MERRA reanalysis product^27^. The precipitation forcing in MERRA-Land merges MERRA precipitation with a gauge-based data product from the National Oceanic and Atmospheric Administration (NOAA) Climate Prediction Center, and the Catchment land surface model used in MERRA-Land is updated to the “Fortuna-2.5” version^27^. MERRA-2 intends to replace the original MERRA reanalysis and ingests important new data types^28^. The Catchment model in MERRA-2 has been updated with rainfall interception and snow model parameters of MERRA-Land, and the precipitation correction is a refined version of MERRA-Land. For MERRA-Land and MERRA-2, there is only one layer for SM from the surface to the bedrock, with “depth-to-rock” depending on local conditions. ST is computed on six vertical soil layers: 0–0.10, 0.10–0.29, 0.29–0.68, 0.68–1.44, 1.44–2.95, and 2.95–12.95 m.
- ERA5 and ERA5-Land
  ERA5 is the fifth generation ECMWF (European Centre for Medium-Range Weather Forecasts) reanalysis of global climate and weather, replacing ERA-Interim^30,31^. Based on a decade of developments in model dynamics and data assimilation, there is a significantly enhanced horizontal resolution (31 km), temporal resolution (hourly) and uncertainty estimation. ERA5 covers 1979–2020 and continues to be updated in near-real-time. ERA5-Land is produced with a finer horizontal resolution of 9 km by running the land component of the ERA5 climate reanalysis but without data assimilation. By March of 2021, the ERA5-Land outputs are only available since 1981. SM and ST are computed on four vertical soil layers (0–0.07, 0.07–0.28, 0.28–1, and 1–2.89 m) for both ERA5 and ERA5-Land.
- GLDAS-Noah v2.0 and GLDAS-Noah v2.1

GLDAS is a global, moderate-resolution (0.25° × 0.25°) offline terrestrial modeling system developed by NASA Goddard Space Flight Center (GSFC) and the NOAA National Centers for Environmental Prediction^29^, thus similar to ERA5. To produce optimal fields of land surface variables in near-real-time, it incorporates satellite- and ground-based observations. GLDAS-Noah drives the Noah land surface model and has two components: one forced with the Princeton meteorological forcing data (i.e. GLDAS-Noah v2.0) and the other forced with a combination of model and observation (i.e. GLDAS-Noah v2.1). GLDAS-Noah v2.0 covers the period 1948–2014, while GLDAS-Noah v2.1 is available from 2000 to the present. There are four vertical layers in the Noah land surface model for both ST and SM: 0–0.1, 0.1–0.4, 0.4–1, and 1–2 m.

### Observation-based wetland/flooded area data

In terms of large uncertainties in current wetland datasets (Table 1) we selected four widely used and available satellite/satellite-based wetland/flooded area data including GIEMS-2 (ref. ^14^), RFW (the Regularly Flooded Wetland map)^10^, WAD2M (a global dataset of Wetland Area and Dynamics for Methane Modeling)^33^, and G2017 (the pantropical wetland extent from an expert system model)^9^ for parameter calibration. Among them, GIEMS-2 and WAD2M include monthly wetland dynamics, while RFW and G2017 are static. The comparison of the four wetland datasets is shown in Supplementary Fig. 1; details on each data are provided below.

- GIEMS-2
  The GIEMS-1 is the first global estimate of monthly inundated areas, derived from passive microwave land surface emissivity^34^. With a 0.25° × 0.25° resolution, GIEMS-1 documents a mean annual maximum inundated area of 9.5 Mkm^2^ for 1993–2007 (including open water, wetlands, and rice paddies, but excluding large lakes), which shows good agreement with existing independent, static inventories as well as regional high-resolution synthetic aperture radar observations^34^. Based on similar retrieval principles with GIEMS-1, GIEMS-2 is developed to less depend on ancillary data with an updated microwave emissivity, and correct a known overestimation over low vegetated areas from GIEMS-1 (ref. ^14^). The period is extended to 1992–2015 for GIEMS-2 and can be updated with the availability of observations. Globally, the mean annual maximum and long-term maximum inundated extent after removing the rice paddies using the Monthly Irrigated and Rainfed Crop Areas dataset (MIRCA2000)^35^ for the period 1992–2015 are 6.7 and 10.9 million km^2^ (hereafter Mkm^2^; sum of mean annual maximum or long-term maximum inundated extent for each grid cell) respectively. The rice paddies are removed here as they are not natural wetlands and cannot be simulated with TOPMODEL.
- RFW
  RFW is a static, high-resolution map (15 arc-sec) of regularly flooded wetlands, developed by overlapping flooded areas (permanent wetlands and flooded vegetation classes) for 2008–2012 from the ESA-CCI land cover map^36^, mean annual maximum inundated areas (including wetlands, rivers, small lakes, and irrigated rice) for 1993–2004 from GIEMS-D15 global inundation extent (downscaled using GIEMS-1)^37^, and long-term maximum surface water areas for 1984–2015 from JRC global surface water bodies product^13^. The large permanent lakes and reservoirs are distinguished using the HydroLAKES database^38^. Globally, RFW covers 9.7% of the land surface area (~13.0 Mkm^2^) including wetlands, river channels, deltas, and flooded lake margins, but excluding large lakes^10^. Due to the mean annual maximum or long-term maximum inundation/surface water extent for 1984–2016 from the three wetland data is used, we treated RFW as the long-term maximum wetland extent in this study. Besides, given that GIEMS-D15 includes artificial rice paddies, we removed them with MIRCA2000 from RFW (~11.9 Mkm^2^ after removing rice paddies).
- WAD2M
  WAD2M dataset used in this study is an improved version of the SWAMPS v3.2 from Jensen *et al*. (ref. ^15^), covering the years 2000 to 2018. With a spatial resolution of 25 km × 25 km, this data was used as input wetland area data of phase 2 of the Global Methane Budget^33^. Given that the initial SWAMPS failed to detect wetlands lacking surface inundation and to differentiate between lakes, wetlands, and other surface water bodies, Zhang *et al*. (ref. ^33^) modified it using a series of independent static wetland distribution data^7,9,39–41^ in an attempt to include missing wetlands under dense canopies. Besides, they removed inland waters (lakes, rivers, and ponds) and rice agriculture with JRC and MIRCA2000, respectively. Globally, the mean annual maximum and long-term maximum wetland extent for the period 2000–2018 estimated by WAD2M are 8.1 Mkm^2^ and 13.2 Mkm^2^ (sum of mean annual maximum or long-term maximum inundated extent for each grid cell) respectively.
- G2017

G2017 (ref. ^9^) is a static, pantropical wetland and peatland extent map (covering 60°S–40°N) at 232 m × 232 m resolution, derived from a hybrid expert model system. With three biophysical indices related to wetland and peat formation (long-term water supply exceeding atmospheric water demand, annually or seasonally waterlogged soils, and geomorphological position where water is supplied and retained), G2017 identifies not only permanently and seasonally wetland areas, but also soil wetness and topographic conditions that favor waterlogging in the absence of flooding for the end of the 20^th^ century. Given the broad coverage of different types of wetlands, we also treated this map as long-term maximum wetland areas. This ‘pantropical’ data (60°S to 40°N) offers the advantage to include non-flooded wetland areas that are missed in satellite-based wetland products. However, note that not all detected wetlands or peatlands in G2017 have been observed. Rice agriculture was also removed with MIRCA2000 from G2017. The resulting wetland and peatland area for 60°S–40°N is 4.0 Mkm^2^.

### The TOPMODEL-based diagnostic model

TOPMODEL as improved by Stocker *et al*. (ref. ^20^) and Xi *et al*. (ref. ^25^) was used to calculate the inundated fraction from WTD at grid-scale in this study. Based on the assumptions that the local hydraulic gradient is approximated by the local topographic slope and the water table variations can be assimilated to a succession of steady states with uniform recharge, the classical TOPMODEL establishes an analytical relationship between the soil moisture deficit and the distributions of local topographic index within a catchment. At grid-scale, the analytical relationship can be represented as:

1 $$CT{I}_{i}-\overline{CT{I}_{x}}=\mathrm{-M}\left({{\Gamma }}_{i}-\overline{{{\Gamma }}_{x}}\right)$$

where CTI indicates the topographic index, defined as the log of the ratio of contributing area to the local slope. We used the CTI data at 500 m × 500 m resolution from Marthews *et al*. (ref. ^22^), where lakes, reservoirs, mountain glaciers, and ice caps are removed using the Global Lakes and Wetlands Database^7^. The $\overline{CT{I}_{x}}$ indicates the average of *CTI*_*i*_ of all sub-grids (index *i*) within the grid cell *x*. *M* indicates a tunable parameter that describes the exponential decrease of soil transmissivity with depth^21^. *Γ*_*i*_ is the water table of the pixel *i* and $\overline{{{\Gamma }}_{{x}}}$ is the mean water table of the grid *x*. When *Γ*_*i*_ is at the soil surface (i.e. *Γ*_*i*_ = 0), the threshold $CT{I}_{x}^{* }$ above which all pixels are flooded for the grid *x* is derived:

2 $$CT{I}_{x}^{* }=\overline{CT{I}_{x}}+{\rm{M}}\cdot \overline{{{\Gamma }}_{x}}$$

The wetlands area is defined as the flooded areas (i.e. *Γ* ≤ 0), the flooded fraction in the grid *x* (*f*_*x*_) being the percentage of pixels with *CTI*_*i*_ larger than a threshold $CT{I}_{x}^{* }$:

$${f}_{x}=\frac{1}{{A}_{x}}{\sum }_{i}{A}_{i}^{* }$$

with

3 $${A}_{i}^{* }=\left\{\begin{array}{c}{A}_{i}\,if\,CT{I}_{i}\ge CT{I}_{x}^{* }\\ 0\,if\,CT{I}_{i} < CT{I}_{x}^{* }\end{array}\right.$$

To reduce the computational costs from the high-resolution CTI data for predicting long time series of wetland area, we used the asymmetric sigmoid function from Stocker *et al*. (ref. ^20^) to fit the “empirical” relationship $\widehat{{\Psi }}$ between $\widehat{f}$ and *Γ*:

4 $${{\rm{\psi }}}_{x}\left({{\Gamma }}_{x}\right)={\left(1+{v}_{x}\cdot {e}^{-{k}_{x}\left({{\Gamma }}_{x}-{q}_{x}\right)}\right)}^{-1/{v}_{x}}$$

where *v*_*x*_, *k*_*x*_, *q*_*x*_ are three parameters of the function. Given a value of parameter *M*, the three parameters can be derived with a sequence of *Γ*_*x*_ spanning a plausible range of values (−1 m to 2 m) and corresponding *f*_*x*_ from the initial TOPMODEL approach (Eq. (3)). Thus, the wetlands in our study are defined as the flooded area simulated by TOPMODEL. As for the range of parameter *M*, Stocker *et al*. (ref. ^20^) used a global uniform value for *M* (*M* = 8) after testing simulated wetland fraction for a range of *M* (7, 8, 9). Nevertheless, given that distinct topography, soil types, and other intrinsic characteristics in different regions, we considered *M* as a tunable, spatially heterogeneous, and grid-specific parameter, with a range of 1–15 following Xi *et al*. (ref. ^25^). Thus, for each grid cell *x* there are 15 choices for *M*, and then 15 sets of (*v*_*x*_, *k*_*x*_, *q*_*x*_). The optimized parameter combination of (*v*_*x*_, *k*_*x*_, *q*_*x*_) is determined by selecting minimum root-mean-square-error (RMSE) between simulated inundated fractions and observations:

5 $$RMSE=\sqrt{\frac{{\sum }_{i=1}^{n}{\left({O}_{i}-{P}_{i}\right)}^{2}}{n}}$$

where *O*_*i*_ and *P*_*i*_ are observed and simulated wetland fraction, respectively. *n* represents the time-series length for wetland extent. For simulations calibrated with RFW and G2017, the RMSE was computed with the long-term maximum (hereafter called MAX) monthly wetland area because the two data sets are static and only record the MAX wetland extent. While for simulations calibrated with GIEMS-2 and WAD2M which include temporal variations of wetland area, we calibrated the parameters with all months, mean seasonal cycle, yearly maximum, and MAX wetland area, but only showed the optimal simulations calibrated with MAX wetland area in this work to keep consistency with RFW and G2017. Besides, to provide more choices for users, we combined all of the four wetland datasets (i.e. the union of long-term maximum wetland extent) to generate a new wetland map (hereafter called MAX_all), and then used the MAX_all to calibrate the parameters to produce seven sets of global wetland extent products with seven soil moisture datasets. The simulations calibrated with yearly maximum wetland area from GIEMS-2 and WAD2M and long-term maximum wetland area from MAX_all are also provided in our resulting products.

Finally, to avoid unrealistically high wetland fraction output from the function, the simulated maximum wetland fraction *f*_*x*_ is constrained by the observed MAX wetland area with a parameter ${f}_{x}^{max}$ (Eq. (6)), which is different from Stocker *et al*. (ref. ^20^). The determination of ${f}_{x}^{max}$ is analyzed in the supplemental material in detail (Supplementary Text 1). Once the value of (*v*_*x*_, *k*_*x*_, *q*_*x*_) are determined, the wetland fraction *f*_*x*_ can be directly derived from the monthly water table *Γ*_*x*_ according to Eqs. (4) and (6).

6 $${f}_{x}=min\left({{\Psi }}_{x}\left({{\Gamma }}_{x}\right),{f}_{x}^{max}\right)$$

### Calculation of water table depth

Water table depth is not computed by land surface models, given their coarse soil vertical discretization. We thus used the saturation deficit of soil moisture (*θ*_*SD*_) as a surrogate of water table depth, *θ*_*SD*_ being defined as an index consisting of saturated volumetric water content and the “actual” soil depth modified by soil freeze/thaw status:

7 $${\theta }_{SD}={z}_{{l}_{0}}-{\sum }_{l=1}^{{l}_{0}}{\theta }_{l}\cdot \frac{\Delta {z}_{l}}{{\theta }_{S}}$$

Subscript *l* represents the *l*^*th*^ soil layer, *l*_0_ is the number of layers above the first frozen soil layer counted from the top (*l* = 1 at the soil surface), *θ*_*l*_ is the monthly volumetric water content in the *l*^*th*^ soil layer (m^3^ m^−3^), $\Delta {z}_{l}$ is the thickness of the *l*^*th*^ soil layer (m), *θ*_*S*_ is the saturated volumetric water content (in m^3^ m^−3^ units, uniform over depth).

As formulated in Eq. (7), ${z}_{{l}_{0}}$ is the thickness of all soil layers (or depth to bedrock) when there is no frozen soil layer. If there exists at least one frozen layer, ${z}_{{l}_{0}}$ is set to the depth of the uppermost frozen soil layer. We excluded the frozen soil layers here given that some important wetland processes such as methane production and transport are insignificant when the soils are frozen. In high latitudes, the presence of frozen soil layers may lead to an overestimation of the wetland fraction due to relatively large *θ*_*SD*_ values even if there is little liquid soil water above the uppermost frozen soil layer. Hence, we used monthly soil temperature (ST) at 70 cm, the Global Record of Daily Landscape Freeze/Thaw Status data^42^, and the Köppen climate classification system^43^ to refine the frozen mask. When the monthly mean ST at 70 cm is below 0 °C, or soil freezing days are more than 5 in a month, or the grid is classified as the Hot desert (BWh) in the Köppen climate classification system, the wetland fraction for the grid is set to zero. However, it should be noted that the algorithm using the ST at 70 cm could omit some unfrozen soil layers above 70 cm, which could lead to bias in estimation of methane emissions from these unfrozen layers. We provided the global wetland maps in our resulting products, but the potential uncertainties in wetland estimation due to the omitted unfrozen layers should be considered, particularly at high latitudes. We used seven reanalysis SM products to compute *θ*_*SD*_ to provide the uncertainty in SM input (Table 2). All data are re-interpolated to 0.25° × 0.25° resolution.

### Evaluation against wetland calibration data and independent satellite products

Although we calibrated parameters of the TOPMODEL-based diagnostic model with the observation-based wetland data, to what extent the simulations can reproduce the spatial patterns and temporal dynamics of the calibration wetland data must be evaluated. For spatial patterns, we calculated the RMSE of wetland area between our simulations and corresponding wetland calibration data following Eq. (5), and evaluated the spatial patterns of simulated wetland extent in two wetland hotspots including Amazon basin and Western Siberia lowlands with three independent global/regional water products. For Amazon basin, we used the global surface water dataset from JRC^13^ (optical satellite images) and the wetland map produced using mosaics of Japanese Earth Resources Satellite (JERS-1) L-band SAR imagery from Hess *et al*. (ref. ^44^, hereafter H2015). For West Siberian lowlands, we used JRC and the Boreal–Arctic Wetland and Lake Dataset (BAWLD, only covers the north of ~55°N) produced using an expert assessment and extrapolated using random forest modelling from climate, topography, soils, permafrost conditions, vegetation, wetlands, and surface water extents and dynamics^45^. For temporal dynamics, since we only used the static wetland area (long-term maximum) from all of the four observation-based wetland products to calibrate parameters, the simulated temporal dynamics can be evaluated with the two dynamic wetland products (GIEMS-2 and WAD2M). Besides, we also used the terrestrial water storage (TWS) from the Gravity Recovery and Climate Experiment (GRACE), which retrieves relative change in TWS from the monthly anomalies of the Earth’s gravity field for 2003–2016 measured by the twin GRACE satellites^46,47^ to evaluate the simulated temporal dynamics.

## Data Records

The global wetland dynamics dataset (GWDD)^48^ produced in this study consists of 28 sets of monthly global/regional wetland extent products derived from seven reanalysis soil moisture data and calibrated with four observation-based wetland products, and can be available at 10.5281/zenodo.4571667. The spatial resolution for the dataset is 0.25° × 0.25°. The temporal coverage of each product is determined by the input soil moisture data in Table 2. These data are stored in NetCDF format with one file per year, defined by three dimensions (lon, lat, and time, indicating longitude, latitude, and month respectively) and a variable (fwet, i.e. wetland fraction).

Naming convention:

fwet_[name of wetland data for calibration]_max_[name of soil moisture data]_reso025_[yyyy].nc ([yyyy] indicates year)

Variable: fwet = wetland fraction [0–1]

## Technical Validation

The evaluation of spatial distribution, seasonal cycle, and interannual variability of wetland extent from the resulting products against corresponding observation-based wetland data and other independent products are shown in the following subsections.

### Spatial distribution of wetland extent

**Fig. 2:**
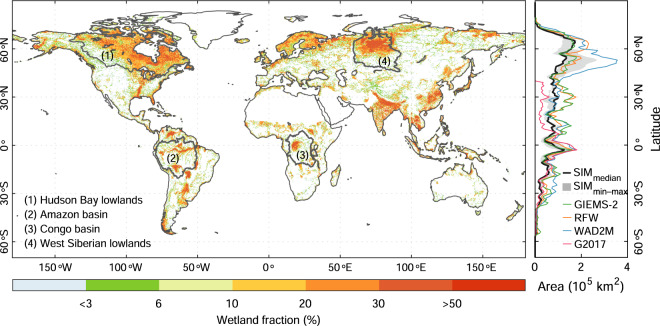
Spatial patterns of wetland extent from the median of our ensemble of simulations. The median simulation was derived using the SM from MERRA-Land and calibrated with RFW wetland map. Four wetland hotspots including Hudson Bay lowlands, Amazon basin, Congo basin, and West Siberian lowlands are highlighted and the 0.25° × 0.25° grids with a <1% wetland fraction from RFW are masked out. The right subset shows zonal wetland area distributions from the median, minimum, maximum wetland area from our ensemble of simulations and four observation-based wetland datasets. Note for all global results, we only used simulations calibrated with GIEMS-2, RFW, and WAD2M, because the lack of coverage in north of 40°N for G2017.

**Fig. 3:**
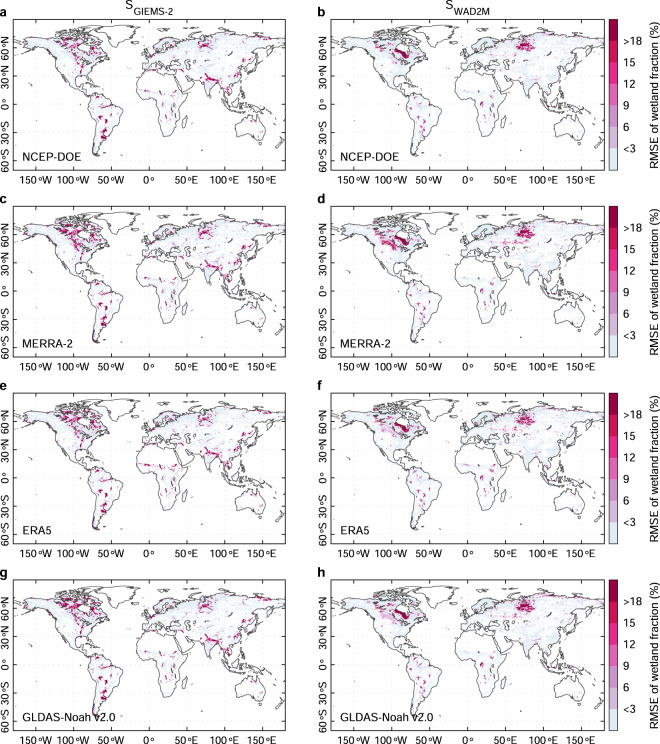
Evaluation of the simulated wetland extent against observations at grid scale. Spatial patterns of root-mean-square-error (RMSE) of long-term maximum wetland extent between observed wetland data and simulations from four soil moisture data including NCEP-DOE, MERRA-2, ERA5, and GLDAS-Noah v2.0, with the parameters calibrated with GIEMS-2 and WAD2M (denoted as S_GIEMS-2_ and S_WAD2M_) respectively. The 0.25° × 0.25° grids with a <1% wetland fraction from RFW are masked out for all maps.

Figure 2 shows the spatial distribution of MAX (long-term maximum) wetland extent from the median of our ensemble of simulations. Globally, the simulated wetland hotspots (wetland fraction >20%) are concentrated in northeastern North America, West Siberian lowlands, South Asia, and Amazon basin. The simulated latitudinal distributions of MAX wetland area reveal the largest peak at 50°N–70°N, and another near the equator. Compared with observation-based wetland products, our ensemble of simulations can reproduce the spatial patterns of observation-based wetland extent (Supplementary Fig. 1) well in most regions with a <3% root-mean-square-error (RMSE) of MAX wetland extent between our ensemble members and corresponding wetland calibration data (Fig. 3 and Supplementary Fig. 2), suggesting the effectiveness of the global implementation of TOPMODEL to simulate wetland dynamics. In some parts of wetland hotspots such as Hudson Bay lowlands and West Siberian lowlands, however, the RMSE is found to be more than 9%, which could be related to the limitation of the SM-based hydrological model in representing wetlands with lateral flow and overland flow contributing water (Supplementary Text 2) and wetlands with frozen soils in the high latitudes (Methods).

**Fig. 4:**
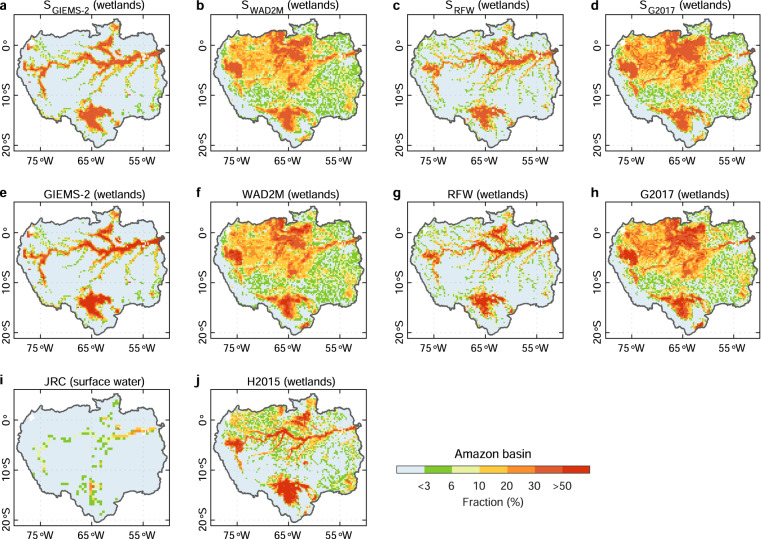
Evaluation of the simulated wetland extent against the corresponding wetland calibration data and independent regional wetland maps for Amazon basin. All simulations showed here (**a**–**d**) are produced based on soil moisture from MERRA-2. The wetland map from JRC (**i**) represents the maximum surface water extent for 1984–2015 from the global surface water dataset from JRC. The wetland map from H2015 (**j**) represents wetlands during the period 1995–1996 for the high-water season from Hess *et al*. (ref. ^44^).

**Fig. 5:**
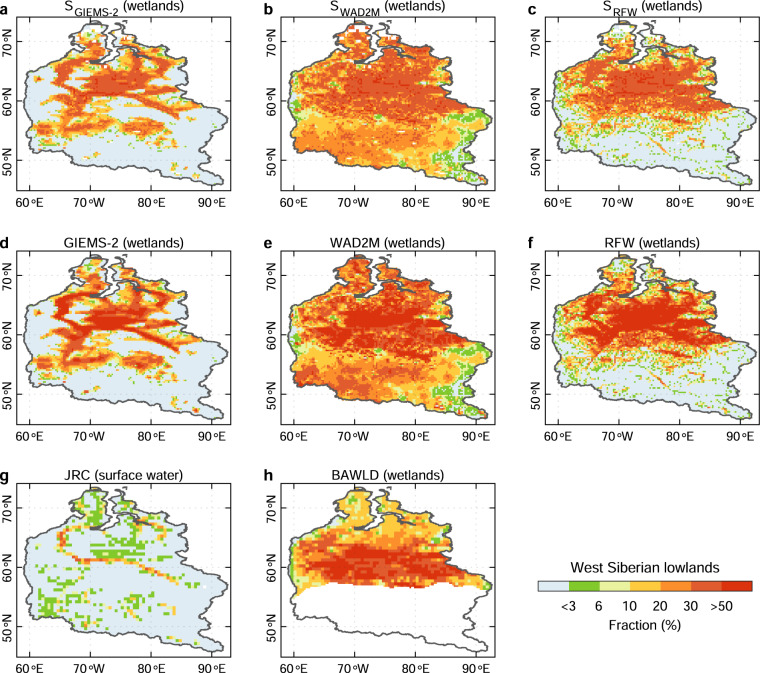
Evaluation of the simulated wetland extent against the corresponding wetland calibration data and independent regional wetland maps for West Siberian lowlands. All simulations showed here (**a**–**d**) are produced based on soil moisture from MERRA-2. The wetland map from JRC (**i**) represents the maximum surface water extent for 1984–2015 from the global surface water dataset from JRC. The wetland map from BAWLD represents wetlands including bog, fen, marsh, and tundra wetland from BAWLD (only covers the north of 55°N).

Regionally, the comparison of simulated wetland extent with the corresponding wetland calibration data and independent global/regional wetland datasets (JRC, H2015, and BAWLD) suggests that our simulations can reproduce the spatial patterns of long-term maximum wetland fraction from the corresponding wetland calibration data in the two wetland hot spots (Amazon basin and West Siberian lowlands), and also show a good consistency with the independent observation-based wetland products (Figs. 4 and 5). However, there are substantial disagreements among simulations calibrated with different observation-based wetland data. By comparing the spatial distributions of wetland extent from simulations using different SM data and different wetland calibration data, we found that the differences in grid-cell wetland fraction among our ensemble of simulations mainly result from the different calibration wetland data (Supplementary Figs. 3 and 4). For example, the simulations based on the same SM data (MERRA-2) but calibrated with different wetland products show a >10% standard deviation (SD) of wetland fraction in most wetland hotspots, against a <3% SD for simulations with the same wetland data (RFW) but different SM data (Supplementary Fig. 5). Hence, hereafter we only show the results from one out of the same reanalysis family (NCEP, MERRA, GLDAS-Noah, or ECWMF) for different calibration wetland observations (simulations calibrated with GIEMS-2, RFW, WAD2M, and G2017 are denoted by S_GIEMS-2_, S_RFW_, S_WAD2M_, and S_G2017_, respectively). Meanwhile, this reminds users of choosing the products they need based on the wetland definition in different wetland calibration data.

### Seasonal cycle of wetland extent

**Fig. 6:**
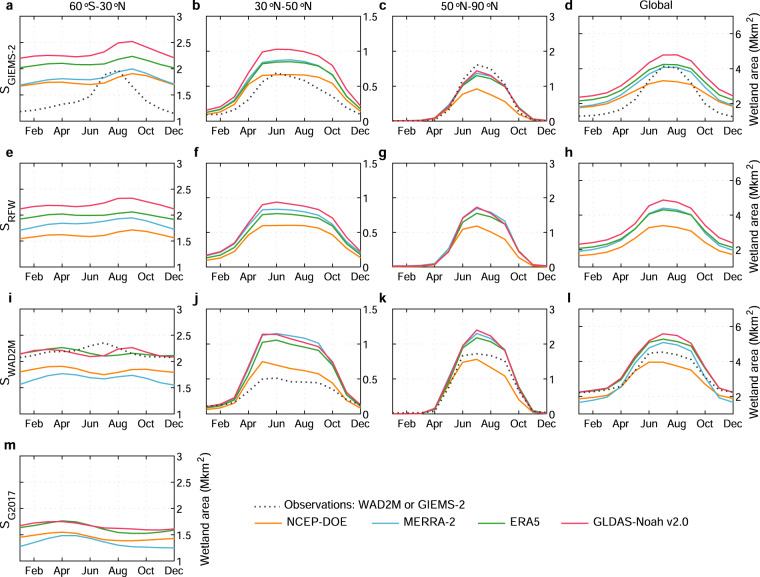
Evaluation of the simulated seasonal cycle of wetland extent against wetland calibration data. Mean seasonal cycle of wetland area from wetland calibration data and simulations based on SM from NCEP-DOE, MERRA-2, ERA5, and GLDAS-Noah v2.0 for global and three latitudinal bands (60° S–30°N, 30°N–50°N, and 50°N–90°N) from 1980 to 2020, with the parameters calibrated with GIEMS-2, RFW, WAD2M, and G2017 (denoted as S_GIEMS-2_, S_RFW_, S_WAD2M_, and S_G2017_), respectively. Note that for GLDAS-Noah v2.0, the time period only covers 1980–2014.

Regarding temporal dynamics of simulated wetland extent, we first present the mean seasonal cycle of wetland area at global scale and for three latitudinal zones (60°S–30°N, 30°N–50°N, and 50°N–90°N) from 1980 to 2020 using four SM data (Fig. 6). Following the sigmoid function established by Stocker *et al*. (ref. ^20^), with a given *M*, the seasonal and interannual variabilities in wetland fraction within a grid are determined by the input SM. Hence, the wetland area at global scale and for the three latitudinal zones shows similar seasonal patterns with SM across all simulations, with a larger seasonal wetland extent in Northern Hemisphere summer and autumn. Regionally, the two northern latitudinal zones (30°N–50°N and 50°N–90°N) dominate the mean seasonal variation of global wetland area, while the region south of 30°N shows a small seasonal variation despite its coverage of 50% of global wetland extents (Table 3) across all ensemble members.

**Table 3:** Estimations of mean annual maximum wetland extent and standard deviation (in Mkm^2^ units) for global and three latitude zones from simulations based on seven soil moisture data, with the parameters calibrated against GIEMS-2, RFW, SWAMPS, and G2017 (denoted as S_GIEMS-2_, S_RFW_, S_SWAMPS_, and S_G2017_) respectively.

Simulations	Soil moisture data	Global		60° S–30°N		30°N–50°N		50°N–90°N	
		Area	SD	Area	SD	Area	SD	Area	SD
S_GIEMS-2_	NCEP-DOE	3.3	0.10	1.9	0.09	0.7	0.02	0.9	0.07
	MERRA-Land	4.3	0.15	2.2	0.11	0.9	0.03	1.3	0.07
	MERRA-2	4.1	0.14	2.0	0.11	0.9	0.03	1.4	0.07
	ERA5	4.3	0.10	2.2	0.07	0.9	0.04	1.3	0.04
	ERA5-Land	4.5	0.10	2.3	0.08	0.9	0.04	1.4	0.04
	GLDAS-Noahv2.0	4.8	0.09	2.5	0.06	1.0	0.03	1.4	0.05
	GLDAS-Noahv2.1	4.7	0.13	2.5	0.11	1.0	0.03	1.4	0.05
S_RFW_	NCEP-DOE	3.4	0.11	1.7	0.06	0.6	0.03	1.2	0.08
	MERRA-Land	4.6	0.15	2.2	0.10	0.9	0.03	1.6	0.09
	MERRA-2	4.4	0.16	2.0	0.10	0.8	0.03	1.7	0.09
	ERA5	4.3	0.09	2.1	0.06	0.8	0.04	1.5	0.05
	ERA5-Land	4.5	0.09	2.1	0.07	0.8	0.03	1.7	0.04
	GLDAS-Noahv2.0	4.9	0.08	2.3	0.05	0.9	0.03	1.7	0.05
	GLDAS-Noahv2.1	4.7	0.13	2.3	0.10	0.9	0.02	1.6	0.06
S_WAD2M_	NCEP-DOE	4.0	0.16	1.9	0.08	0.7	0.07	1.6	0.11
	MERRA-Land	5.3	0.19	2.1	0.11	1.2	0.04	2.2	0.11
	MERRA-2	5.1	0.17	1.8	0.10	1.2	0.04	2.3	0.11
	ERA5	5.3	0.12	2.3	0.07	1.1	0.05	2.2	0.06
	ERA5-Land	5.6	0.15	2.3	0.09	1.1	0.06	2.4	0.05
	GLDAS-Noahv2.0	5.6	0.11	2.3	0.06	1.2	0.03	2.4	0.07
	GLDAS-Noahv2.1	5.3	0.17	2.2	0.14	1.1	0.04	2.2	0.08
S_G2017_	NCEP-DOE	—	—	1.6	0.07	—	—	—	—
	MERRA-Land	—	—	1.7	0.09	—	—	—	—
	MERRA-2	—	—	1.5	0.09	—	—	—	—
	ERA5	—	—	1.8	0.06	—	—	—	—
	ERA5-Land	—	—	1.8	0.07	—	—	—	—
	GLDAS-Noahv2.0	—	—	1.8	0.04	—	—	—	—
	GLDAS-Noahv2.1	—	—	1.8	0.09	—	—	—	—

Note that the global and regional wetland area shown here are computed as mean annual maximum of global/regional total wetland extent, which is smaller than that computed at grid scale in the Methods section. Besides, S_G2017_ only cover 60°S–40°N.

The observation-based wetland data with temporal dynamics show similar seasonal patterns with our ensemble of simulations, but with a higher estimate (~0.5 Mkm^2^) in some months across three latitudinal zones (Fig. 6). Please note that the temporal dynamics of wetland area from wetland calibration data and our simulations are comparable because the results shown in Fig. 6 are calibrated with the MAX wetland area (that is, only at one epoch), but not constrained by the temporal variations of observed wetland data. The biased estimation in some months is mainly attributed to that the optimized parameter *M* in TOPMODEL is relaxed in “non-MAX” months to match the observed MAX wetland fraction in terms of the notable underestimation of observed MAX wetland extent in most wetland hotspots (Figs. 2–3; Supplementary Figs. 6–7). When calibrated with wetland extent with temporal dynamics, the simulated absolute wetland area is closer to WAD2M and GIEMS-2 (Supplementary Figs. 8–9). To satisfy the need for the more accurate absolute value of monthly wetland extent for S_GIEMS-2_ and S_WAD2M_, we provided additional simulations calibrated with yearly maximum wetland area in our resulting products. Moreover, we also compared the simulated and observed month of annual maximum wetland extent at grid scale. Throughout the globe, our ensemble of simulations can reproduce spatial patterns of the observed month of annual maximum wetland extent basically (Supplementary Fig. 10–11).

### Interannual variability of wetland extent

**Fig. 7:**
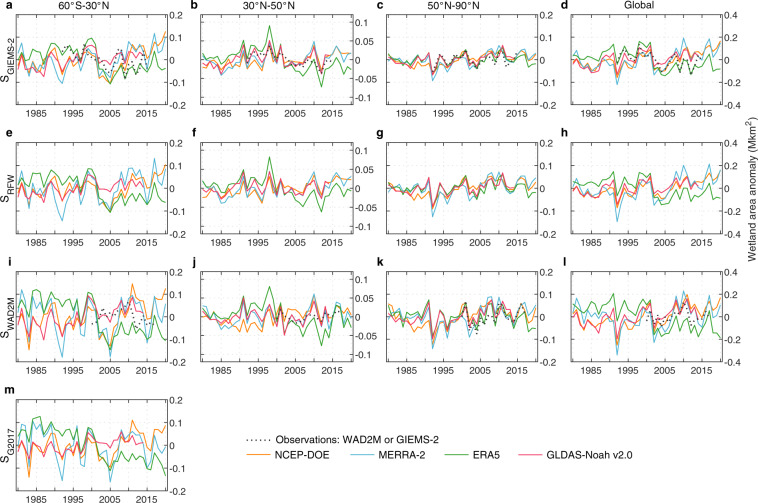
Evaluation of the simulated interannual variabilities of wetland extent against wetland calibration data. Interannual variability in wetland area from wetland calibration data and simulations based on SM from NCEP-DOE, MERRA-2, ERA5, and GLDAS-Noah v2.0 for global and three latitudinal bands (60° S–30°N, 30°N–50°N, and 50°N–90°N) for the period 1980–2020, with the parameters calibrated with GIEMS-2, RFW, WAD2M, and G2017 (denoted as S_GIEMS-2_, S_RFW_, S_WAD2M_, and S_G2017_), respectively. For GLDAS-Noah v2.0, the time period only covers 1980–2014.

Interannual variability (IAV) of wetland area during the period 1980–2020 at global scale and three latitude zones (60°S–30°N, 30°N–50°N, and 50°N–90°N) is displayed in Fig. 7 from GIEMS-2, WAD2M, and our ensemble of simulations. Despite the substantial disagreements in input SM data and wetland observation data, the IAV of global wetland area shows a good agreement between different ensemble members, with a significantly positive correlation across ~60% of simulations (R = 0.32–0.99, p < 0.05). Owing to a discrepant IAV of wetland area since 2008, however, simulated wetland area using SM from GLDAS-Noah v2.1, ERA5, and ERA5-Land show a weak positive correlation with the other four SM data. Influenced by different wetland observations, the IAV of global wetland area across simulations calibrated with different observation data show some discrepancies, with a SD of 0.07–0.11 Mkm^2^, 0.06–0.11 Mkm^2^, and 0.08–0.15 Mkm^2^ for S_GIEMS-2_, S_RFW_, and S_WAD2M_ respectively. Regionally, the simulated IAV of global wetland area is mainly contributed by regions south of 30 ° N (54–84%, 51–90%, 61–88% for S_GIEMS-2_, S_RFW_, and S_WAD2M_) and boreal regions (29–38%, 36–48%, 31–47% for S_GIEMS-2_, S_RFW_, and S_WAD2M_).

**Fig. 8:**
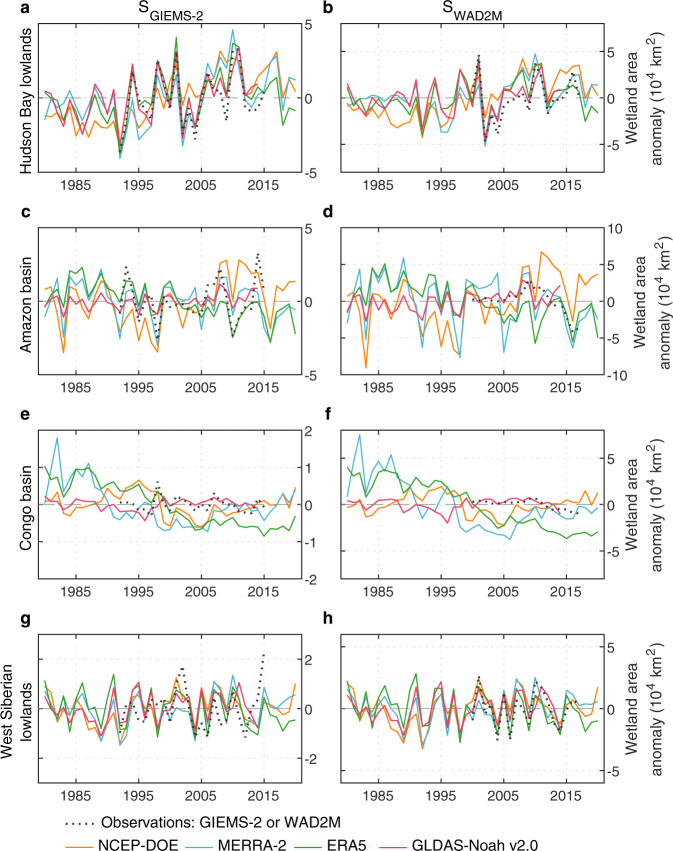
Evaluation of the simulated interannual variabilities of wetland extent against wetland calibration data at basin scale. Interannual variability in wetland area from GIEMS-2, WAD2M, and simulations based on SM from NCEP-DOE, MERRA-2, ERA5, and GLDAS-Noah v2.0 for Hudson Bay lowlands, Amazon basin, Congo basin, and West Siberian lowlands for the period 1980–2020, with the parameters calibrated with GIEMS-2 and WAD2M (denoted as S_GIEMS-2_ and S_WAD2M_), respectively. The spatial locations of the four wetland hotspots are shown in Fig. 1. For GLDAS-Noah v2.0, the time period only covers 1980–2014.

Even though the parameters were calibrated with observed wetland extent without temporal variations (MAX), the IAV in global wetland area from most simulations shows significantly positive correlations with observed wetland area from both GIEMS-2 (R = 0.19–0.79, p < 0.05; Table 4) and WAD2M (R = 0.50–0.70, p < 0.05). This indicates that our variant of TOPMODEL can capture the observed IAV of wetland area well (Fig. 7). Among the seven SM data, simulations from ERA5 (R = 0.79, p < 0.05) and ERA5-Land (R = 0.77, p < 0.05) reproduce the best IAV in GIEMS-2 wetland area while simulations from MERRA-Land (R = 0.70, p < 0.05) and MERRA-2 (R = 0.66, p < 0.05) are more consistent with WAD2M (Table 4). Regionally, almost all simulations show significantly positive correlations with observed IAV of wetland area in temperate regions (R = 0.36–0.90 and 0.26–0.54 for GIEMS-2 and WAD2M, p < 0.1) and boreal regions (R = 0.59–0.88 and 0.55–0.86 for GIEMS-2 and WAD2M, p < 0.05). In regions south of 30 ° N, the simulated IAV of wetland area based on SM from ERA5 and ERA5-Land is significantly correlated with GIEMS-2 (R = 0.60–0.63, p < 0.05), while WAD2M has insignificant or even negative correlations with all simulations (Table 4). More detailed comparison can be found in Supplementary Text 3 and Supplementary Table 1. The positive correlations between our simulations with GIEMS-2 or WAD2M can also be found at basin scale (Fig. 8). At grid scale, our simulated SD of global wetland area is close to GIEMS-2, but shows an obvious overestimation (~0.04 Mkm^2^, ~70%) relative to WAD2M owing to the very small interannual variations across most regions for WAD2M (Supplementary Figs. 12–13).

**Table 4:** Correlations of wetland area between GIEMS-2 or SWAMPS and the corresponding simulations for global and three latitude zones.

Simulations	Soil moisture data	Correlation coefficient			
		Global	60°S–30°N	30°N–50°N	50°N–90°N
S_GIEMS-2_ (1992–2014)	NCEP-DOE	0.31	0.21	0.36*	0.59**
	MERRA-Land	0.19	−0.13	0.65**	0.67**
	MERRA-2	0.24	−0.02	0.53**	0.66**
	ERA5	0.79**	0.63**	0.90**	0.88**
	ERA5-Land	0.77**	0.60**	0.87**	0.86**
	GLDAS-Noahv2.0	0.49**	0.17	0.73**	0.75**
	GLDAS-Noahv2.1	0.65**	0.42	0.72**	0.71**
S_WAD2M_ (2000–2014)	NCEP-DOE	0.60**	−0.20	0.39	0.58**
	MERRA-Land	0.70**	−0.05	0.51*	0.75**
	MERRA-2	0.66**	−0.08	0.45*	0.55**
	ERA5	0.50*	−0.38	0.46*	0.86**
	ERA5-Land	0.52**	−0.41	0.54**	0.80**
	GLDAS-Noahv2.0	0.65**	−0.04	0.52**	0.69**
	GLDAS-Noahv2.1	0.60**	−0.01	0.26	0.78**

*p < 0.1, **p < 0.05.

**Fig. 9:**
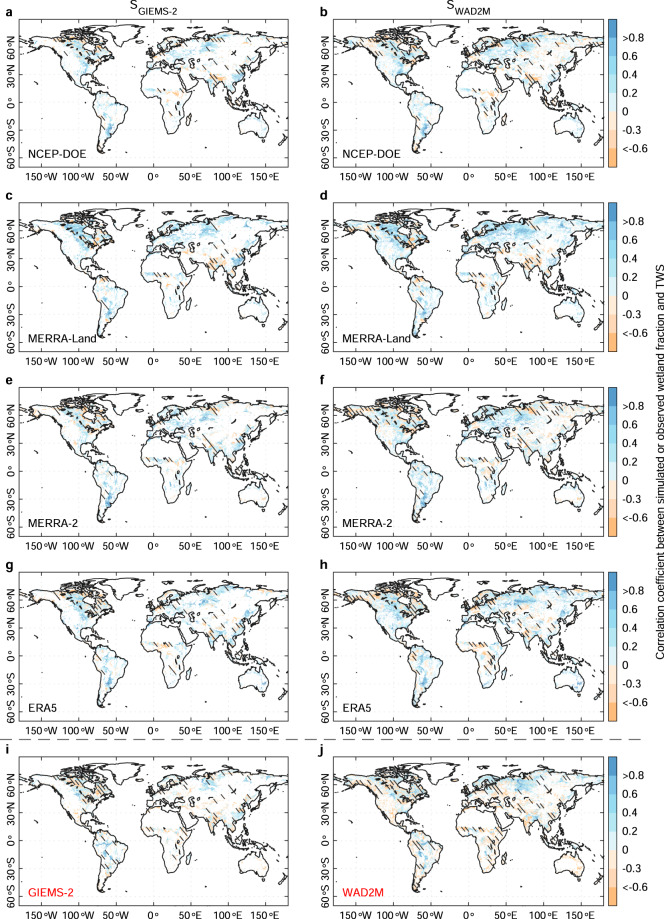
Evaluation of the simulated wetland extent against terrestrial water storage (TWS) from GRACE. Spatial distributions of correlations between TWS from GRACE and wetland fraction from GIEMS-2, WAD2M, and simulations based on four soil moisture data including NCEP-DOE, MERRA-2, ERA5, and GLDAS-Noah v2.0 for 2003–2016, with the parameters calibrated with GIEMS-2 and WAD2M (denoted as S_GIEMS-2_ and S_WAD2M_) respectively. The 0.25° × 0.25° grids with a <1% wetland fraction from RFW are masked out for all maps. The black hatching indicates the correlations are statistically significant (p < 0.05).

In addition to the observed wetland data, we also evaluated the interannual variabilities of simulated mean annual wetland extent against the terrestrial water storage (TWS) for 2003–2016 from GRACE satellites^46,47^. As shown in Fig. 9 and Supplementary Fig. 14, different ensemble members consistently show a positive correlation with the TWS anomaly (the median of R = 0.19–0.33) across 70–80% wetland grids (wetland fraction >1% at 0.25° × 0.25° spatial resolution), especially in global wetland hotspots such as West Siberian lowlands, North America, and South America. Disagreements of temporal variations between wetland extent and TWS are mainly found in some arid regions such as Africa and Central Asia, where have few wetlands. This implies the IAV of the simulated wetland extent from these reanalysis data can present a very good agreement with the TWS from GRACE.

## Usage Notes

We provide 28 sets of monthly global/regional wetland extent products but users can choose the group of simulations they want based on the wetland definitions of different wetland calibration data. Among seven SM inputs, the optimal simulation is suggested to be the one using SM which reproduces the interannual variability of observation-based wetland data best. Due to the omitted unfrozen soil layers from our algorithm for water table depth, we note the potential uncertainties in wetland simulation over high latitudes. Moreover, to satisfy the need for the more accurate absolute value of monthly wetland extent for S_GIEMS-2_ and S_WAD2M_, we provided additional simulations calibrated with yearly maximum wetland area. To provide more choice for users, we also provided additional simulations calibrated with the union of long-term maximum wetland extent (MAX_all) in our resulting products.

## Supplementary information

Supplementary Information

## Data Availability

Computer codes to fit the parameters of the TOPMODEL-based diagnostic model are publicly available on GitHub (https://github.com/yixixy/Wetland_simulation_by_TOPMODEL)^49^.
