# Author Correction: Gridded maps of wetlands dynamics over mid-low latitudes for 1980–2020 based on TOPMODEL

**DOI:** 10.1038/s41597-022-01588-9

**Published:** 2022-08-02

**Authors:** Yi Xi, Shushi Peng, Agnès Ducharne, Philippe Ciais, Thomas Gumbricht, Carlos Jimenez, Benjamin Poulter, Catherine Prigent, Chunjing Qiu, Marielle Saunois, Zhen Zhang

**Affiliations:** 1 Sino-French Institute for Earth System Science, Laboratory for Earth Surface Processes, College of Urban and Environmental Sciences, and, Peking University, Beijing, 100871 China; 2 Sorbonne Université, CNRS, EPHE, Laboratoire METIS (Milieux environnementaux, transferts et interaction dans les hydrosystèmes et les sols), 75005 Paris, France; 3 Laboratoire des Sciences du Climat et de l’Environnement, LSCE/IPSL, CEA-CNRS-UVSQ, Université Paris-Saclay, 91191 Gif-sur-Yvette, France; 4 Center for International Forestry Research (CIFOR), Bogor, Indonesia; 5 Karttur AB, Stockholm, Sweden; 6 Estellus, Paris, France; 7 Sorbonne Université, Observatoire de Paris, Université PSL, CNRS, LERMA, Paris, France; 8 NASA Goddard Space Flight Center, Biospheric Science Laboratory, Greenbelt, MD 20771 USA; 9 Department of Geographical Sciences, University of Maryland, College Park, USA

**Keywords:** Hydrology, Freshwater ecology, Limnology

Correction to: *Scientific Data* 10.1038/s41597-022-01460-w, published online 18 June 2022

The citations for the ERA data in this paper were incorrect in the original version at references 30 and 31.

The original references were:

30. Dee, D. P. *et al*. The ERA-Interim reanalysis: configuration and performance of the data assimilation system. *Q. J. Roy. Meteor. Soc.* **137**, 553–597 (2011).

31. Hersbach, H. *et al*. Goodbye ERA-Interim. *Hello ERA5. ECMWF Newsletter* **159**, 17–24 (2019).

The correct references are

30. Muñoz-Sabater, J. ERA5-Land hourly data from 1981 to present. *Copernicus Climate Change Service (C3S) Climate Data Store (CDS)*. 10.24381/cds.e2161bac (2019).

31. Muñoz-Sabater, J. *et al*. ERA5-Land: A state-of-the-art global reanalysis dataset for land applications. *Earth Syst. Sci. Data* **13**, 4349–4383, 10.5194/essd-13-4349-2021 (2021).

This has been corrected in the published article.

