# Supplementary material for: Gridded maps of wetlands dynamics over mid-low latitudes for 1980–2020 based on TOPMODEL
Source: Sci Data. 2022 Jun 18;9:347. doi: 10.1038/s41597-022-01460-w (PMC9206665; doi:10.1038/s41597-022-01460-w)

Supplementary Information of

Gridded maps of wetlands dynamics over mid-low latitudes for 1980–2020 based on TOPMODEL

Yi Xi[1], Shushi Peng[1], Agnès Ducharne[2], Philippe Ciais[3], Thomas Gumbricht[4,5], Carlos Jimenez[6,7], Benjamin Poulter[8], Catherine Prigent[7,6], Chunjing Qiu[3], Marielle Saunois[3], and Zhen Zhang[9]

1. Sino-French Institute for Earth System Science, Laboratory for Earth Surface Processes, College of Urban and Environmental Sciences, and, Peking University, Beijing 100871, China
2. Sorbonne Université, CNRS, EPHE, Laboratoire METIS (Milieux environnementaux, transferts et interaction dans les hydrosystèmes et les sols), 75005 Paris, France
3. Laboratoire des Sciences du Climat et de l'Environnement, LSCE/IPSL, CEA-CNRS-UVSQ, Université Paris-Saclay 91191 Gif-sur-Yvette, France
4. Center for International Forestry Research (CIFOR), Bogor, Indonesia
5. Karttur AB, Stockholm, Sweden
6. Estellus, Paris, France
7. Sorbonne Université, Observatoire de Paris, Université PSL, CNRS, LERMA, Paris, France
8. NASA Goddard Space Flight Center, Biospheric Science Laboratory, Greenbelt, MD 20771, USA
9. Department of Geographical Sciences, University of Maryland, USA

corresponding author: Shushi Peng (speng@pku.edu.cn)

Contents of this file:

Supplementary Text 1 to 3
Supplementary Table 1
Supplementary Figures 1 to 19

Supplementary Text 1. The determination of the parameters constraining maximum wetland fraction.

In the framework of Stocker et al. (ref. [1]), parameter CTI_{min} indicates a CTI threshold for flooding regardless of the water table depth and it limits simulated maximum wetland fraction from the asymmetric sigmoid function (see their Eqs. (3) and (4)). While in our study, the maximum wetland fraction is directly constrained by long-term maximum wetland extents from wetland calibration data following Xi et al. (ref. [2]), and thus CTI_{min} is redundant and removed here. Taking a wetland calibration data (RFW) and a reanalysis soil moisture dataset (MERRA-2) as example, we concluded three possible cases when simulating only with the tunable M (1–15). As formulated in Eqs. (1)–(3), simulated wetland fraction gets the maximum value when $M = 1$ and gets the minimum value when $M = 15$. The three possible cases are summarized as follows:

Case 1: simulated MAX wetland fraction is always smaller than MAX wetland extent from the corresponding wetland calibration data.

Case 2: simulated maximum MAX wetland fraction when $M = 1$ is larger but when $M = 15$ is smaller than wetland extent from calibration data.

Case 3: simulated MAX wetland fraction is always larger than MAX wetland extent from the corresponding wetland calibration data.

In Supplementary Fig. 15, we presented the spatial pattern of the comparison of MAX wetland extent between RFW and the optimized simulation only with tunable M based on MERRA-2 SM data. Globally, there are 14.7%, 38.4%, and 46.9% grids for the three cases respectively. For Case 1 (Supplementary Fig. 15b), simulated wetland fraction with the MAX θ_{SD} from MERRA-2 is less than that from RFW even when $M = 1$. This underestimation of wetland extent from RFW could be associated with the underestimated SM in the wetland hotspots (especially where surface water is omitted or underestimated in these reanalysis SM data) and / or the disagreements of instinct characteristics between SM and natural wetlands, and / or the limitation of the asymmetric sigmoid function in Eq. (4). If the accuracy of the wetland fraction from RFW can be referenced, extensive wetlands located in northeastern North America, West Siberian lowlands, and Amazon basin (Supplementary Fig. 1) could be underestimated in our products. For Case 2 (Supplementary Fig. 15c), an optimal M can be found to match the MAX wetland fraction from RFW even without any other constraint. For Case 3 (Supplementary Fig. 15d), even when $M = 15$ simulated MAX wetland fraction is still larger than RFW, and in this case the parameter f_x^{max} should play a part in constraining simulated MAX wetland fraction. Stocker et al. (ref. [1]) computed the parameter f_x^{max} using CTI_{min} as the CTI threshold of the grid instead of CTI_x^* and then reduced the simulated wetland extent for grids for Case 3. In this study, we used the maximum wetland fraction from observation-based wetland data as f_x^{max} directly to constrain MAX wetland fraction in Case 3, and thus the redundant parameter CTI_{min} was removed. Nevertheless, the simulated absolute value of wetland fraction still should be treated carefully because constraining using wetland calibration data means we neglect the biases in these data sets, and the time series of observation-constrained wetland extent could deviate from the temporal dynamics of the input SM. Overall, the wetland simulation using the diagnostic model calls for more accurate calibration data and input SM data.

Supplementary Text 2. Uncertainties of the simulated global wetland products.

The uncertainties of our ensemble simulations mainly stem from the input SM, the choice of calibration wetland data and the calibration methods, and the structural uncertainties in TOPMODEL.

1) Input SM data

Limited by the availability of SM only for shallow (0–5 cm) soil layer and the discontinuity in space for current satellite-based SM data[4], we used seven reanalysis SM products with high spatiotemporal resolution as the input of the diagnosed model. Different reanalysis SM datasets provide the uncertainties in wetland simulation, and also make it possible to update the global wetland dynamics in real-time. Although the accuracy of these SM products has been validated against a wealth of ground-based in situ observations[5-9] and independent remote sensing products[10-12], there are still notable disagreements in the absolute value and spatial pattern of SM estimation across wetland grids between different reanalysis products, even for the sibling SM data from the same reanalysis family (Supplementary Fig. 16). Relative to different wetland products, however, the discrepancies in SM contribute very small uncertainties (<3%) of simulated global wetland extent (Supplementary Fig. 5). Moreover, note that the surrogate of WTD with the saturation deficit of soil moisture in this study misses surface water, which would lead to the underestimation in real WTD across very wet grids. In terms of the underestimation of MAX wetland fraction from observation-based wetland data in wetland hotspots, the underestimation of WTD could result in the over-calibration of parameter M. Regarding the seasonal and interannual variabilities of the input SM, we evaluated the temporal variations in mean SM across all soil layers against TWS (terrestrial water storage) from GRACE (Supplementary Fig. 17). Seven SM data consistently show a positive correlation with the TWS anomaly across nearly 80% of wetland grids, especially in global wetland hotspots. Among seven SM data, ERA5-Land shows the best consistency with GRACE.

2) Choice of wetland calibration data and the calibration methods

Table 1 lists different water / inundation 'calibration' datasets including static and dynamic, global and regional, but with considerable discrepancies in wetland area estimation. We chose four widely used global / regional wetland products for parameter calibration, covering a MAX wetland area of 10.9, 11.9, 13.2, and 4.0 (only for 60°S–40°N) Mkm^2 respectively. Among them, RFW and G2017 are static but potentially include saturated but non-flooded area[13,14]; GIEMS-2 is dynamic but misses small wetlands[15]; WAD2M from Zhang et al. (ref. [16]) modifies the initial SWAMPS[17] using static wetland data to include some missing wetlands, but changes the initial temporal dynamic concurrently. In the initial TOPMODEL, wetlands are defined as flooded areas with a WTD above the soil surface, but due to the adjustment of parameter M in each grid to match the observed MAX wetland area, the wetland extent from our simulations is largely dependent on the choice of the wetland dataset used for calibration. As shown in Supplementary Figs. 6–7, the optimized parameter M shows a sensitive response to the change in wetland calibration data but a subtle response to the change in soil moisture data (Supplementary Figs. 6–7). Thus, compared to the input SM data, more uncertainties in

wetland simulation come from the choice of wetland data. As for the calibration methods, intuitively, calibrated with the dynamic observed wetlands should be the optimal choice to reproduce the absolute value and temporal dynamics of observed wetland extent. The static wetland products limit the calibration with long-term wetland extent. For GIEMS-2 and WAD2M which have temporal dynamics, the simulations calibrated with MAX wetland area present some overestimates of observed wetland area, relative to simulations calibrated with long time series of wetland area. Nevertheless, due to the input SM determining temporal variations in the simulated wetland area, simulations with different calibration methods show few differences in seasonal pattern and IAV of wetland area (Supplementary Figs. 8–9, 18–19). The comparison of interannual variations in wetland area from the amplitude and correlations shows that the simulations from ERA5 and ERA5-Land can reproduce GIEMS-2 best, while the simulation from GLDAS-Noah v2.0 is more consistent with WAD2M (Supplementary Table 1; Supplementary Text 3).

3) TOPMODEL

Due to the availability of CTI at high resolution, TOPMODEL keeps on being popular to simulate wetlands. Yet, limitations still exist when depicting wetland dynamics using the model. First, even neglecting a series of simplifying assumptions established in TOPMODEL, the CTI threshold for flooded in TOPMODEL (Methods) could not be a perfect surrogate for wetlands everywhere, because the wetting or dry for some grids does not rely on the mean WTD and the CTI in some sub-grids. Thus, Stocker et al. (ref. [1]) used a cutoff value CTI_{min} to constrain the wetland fraction derived from TOPMODEL. Besides, due to TOPMODEL only assumes all water comes from local grid cell, it fails to well simulate wetlands fed by upstream discharge or floodplains in the tropics. Some important wetland types such as floodplains, peatlands, and thermokarst lakes are missed in TOPMODEL[18,19]. Moreover, the removal of rice paddies and lakes with the static products in our study could also propagate uncertainties in the simulation of global wetland dynamics.

Supplementary Text 3. Further comparison of interannual variations of simulations and observation-based wetland data.

For simulations calibrated with GIEMS-2 and WAD2M, we further evaluated the simulated interannual variability (IAV) of wetland area against observation-based wetland data using the squared differences between standard deviations (SDSD) and the lack of correlation weighted by the standard deviations (LCS) respectively as Kobayashi et al. (ref. [3]):

$$SDSD = (SD_S - SD_O)^2 \tag{S1}$$

$$\mathrm{LCS} = 2SD_S SD_O(1 - r) \tag{S2}$$

where the standard deviation of observation-based and simulated wetland area is denoted as SD_O and SD_S, and the Pearson's correlation coefficient between the simulation and observation-based wetland data is denoted as r, namely $SD_S = \sqrt{\frac{1}{n}\sum_{i=1}^{n}(x_i - \bar{x})^2}$, $SD_O = \sqrt{\frac{1}{n}\sum_{i=1}^{n}(y_i - \bar{y})^2}$, $r = \left[\frac{1}{n}\sum_{i=1}^{n}(x_i - \bar{x})(y_i - \bar{y})\right]/(SD_S SD_O)$. The two metrics are decomposed from the metric of mean squared deviation (MSE), which can be used to evaluate the biases between observation-based and simulated IAV of wetland area[3].

As shown in Supplementary Table 1, for GIEMS-2, due to all simulations having a small (less than 12 $\times 10^4$ km^2) SDSD, the biases with wetland area from GIEMS-2 are determined by the correlations with GIEMS-2. Thus, the best soil moisture data for GIEMS-2 determined with the sum of SDSD and LCS (the lack of correlation weighted by the standard deviations), i.e. ERA5 and ERA5-Land are consistent with those determined with the Pearson's correlation coefficient in the "Technical Evaluation" section. While for WAD2M, except for the simulations from GLDAS Noah v2.0, all simulations show a larger than 10 $\times 10^4$ km^2 SDSD against wetland area from WAD2M. As a result, the LCS (weighted by the standard deviations) between simulated wetland area with WAD2M shows that simulations from GLDAS-Noah v2.0 and NCEP-DOE reproduce the best amplitude and variations of wetland area from WAD2M.

Reference

1. Stocker, B. D., Spahni, R. & Joos, F. DYPTOP: a cost-efficient TOPMODEL implementation to simulate sub-grid spatio-temporal dynamics of global wetlands and peatlands. *Geosci. Model Dev.* **7**, 3089-3110, (2014).
2. Xi, Y., Peng, S., Ciais, P. & Chen, Y. Future impacts of climate change on inland Ramsar wetlands. *Nat. Clim. Chang.* **11**, 45-51, (2021).
3. Kobayashi, K. & Salam, M. U. Comparing Simulated and Measured Values Using Mean Squared Deviation and its Components. *Agron. J.* **92**, 345-352, (2000).
4. Dorigo, W. A. *et al.* Evaluation of the ESA CCI soil moisture product using ground-based observations. *Remote Sens. Environ.* **162**, 380-395, (2015).
5. Lu, C.-H. *et al.* Evaluation of Soil Moisture in the NCEP–NCAR and NCEP–DOE Global Reanalyses. *J. Hydrometeorol.* **6**, 391-408, (2005).
6. Dorigo, W. A. *et al.* The International Soil Moisture Network: a data hosting facility for global in situ soil moisture measurements. *Hydrol. Earth Syst. Sci.* **15**, 1675-1698, (2011).
7. Reichle, R. H. *et al.* Assessment and Enhancement of MERRA Land Surface Hydrology Estimates. *J. Climate* **24**, 6322-6338, (2011).
8. Aihui, W. & Xubin, Z. Evaluation of multireanalysis products with in situ observations over the Tibetan Plateau. *J. Geophys. Res.-Atmos.* **117**, D05102, (2012).
9. Bi, H., Ma, J., Zheng, W. & Zeng, J. Comparison of soil moisture in GLDAS model simulations and in situ observations over the Tibetan Plateau. *J. Geophys. Res.-Atmos.* **121**, 2658-2678, (2016).
10. Syed, T. H., Famiglietti, J. S., Rodell, M., Chen, J. & Wilson, C. R. Analysis of terrestrial water storage changes from GRACE and GLDAS. *Water Resour. Res.* **44**, (2008).
11. Wang, W., Cui, W., Wang, X. & Chen, X. Evaluation of GLDAS-1 and GLDAS-2 Forcing Data and Noah Model Simulations over China at the Monthly Scale. *J. Hydrometeorol.* **17**, 2815-2833, (2016).
12. Reichle, R. H. *et al.* Assessment of MERRA-2 Land Surface Hydrology Estimates. *J. Climate* **30**, 2937-2960, (2017).
13. Gumbricht, T. *et al.* An expert system model for mapping tropical wetlands and peatlands reveals South America as the largest contributor. *Global Change Biol.* **23**, 3581-3599, (2017).

14 Tootchi, A., Jost, A. & Ducharne, A. Multi-source global wetland maps combining surface water imagery and groundwater constraints. *Earth Syst. Sci. Data* **11**, 189-220, (2019).

15 Prigent, C., Jimenez, C. & Bousquet, P. Satellite-derived global surface water extent and dynamics over the last 25 years (GIEMS-2). *J. Geophys. Res.-Atmos.* **125**, (2019).

16 Zhang, Z. *et al.* Development of a global dataset of Wetland Area and Dynamics for Methane Modeling (WAD2M). *Earth Syst. Sci. Data Discuss.* **2020**, 1-50, (2020).

17 Jensen, K. & Mcdonald, K. Surface Water Microwave Product Series Version 3: A Near-Real Time and 25-Year Historical Global Inundated Area Fraction Time Series From Active and Passive Microwave Remote Sensing. *IEEE Geosci. Remote S.* **16**, 1402-1406, (2019).

18 Schuur, E. A. G. *et al.* Climate change and the permafrost carbon feedback. *Nature* **520**, 171-179, (2015).

19 Tan, Z. & Zhuang, Q. Methane emissions from pan-Arctic lakes during the 21st century: An analysis with process-based models of lake evolution and biogeochemistry. *J. Geophys. Res-Biogeo.* **120**, 2641-2653, (2015).

Supplementary Table 1. Comparison of interannual variability in wetland area from GIEMS-2 or WAD2M and the corresponding simulations for global and three latitude zones. The squared differences between standard deviations and the lack of correlation weighted by the standard deviations are denoted as SDSD and LCS respectively, in the unit of 10^4 km^2. The SUM indicates the sum of SDSD and LCS.

Simulation	Soil moisture data	Global			60°S–30°N			30°N–50°N			50°N–90°N		
		SDSD	LCS	SUM	SDSD	LCS	SUM	SDSD	LCS	SUM	SDSD	LCS	SUM
$S_{GIEMS-2}$ (1992–2014)	NCEP-DOE	0.4	67.5	67.9	0.6	34.0	34.7	0.0	3.8	3.9	0.2	5.7	5.8
	MERRA-Land	11.4	109.5	121.0	3.1	57.5	60.6	0.4	2.6	3.0	2.4	6.4	8.8
	MERRA-2	5.1	91.2	96.4	0.5	43.1	43.6	0.2	3.2	3.3	1.5	6.1	7.7
	ERA5	8.0	26.4	34.4	2.6	18.3	20.9	4.2	1.2	5.4	0.1	1.7	1.8
	ERA5-Land	6.1	27.9	34.0	2.5	19.7	22.2	2.6	1.4	3.9	0.1	1.9	2.0
	GLDAS-Noah v2.0	0.3	42.0	42.2	1.6	20.8	22.5	0.2	1.9	2.0	0.1	3.3	3.4
	GLDAS-Noah v2.1	0.9	32.3	33.3	1.2	23.2	24.4	0.9	1.6	2.5	0.1	3.4	3.5
S_{WAD2M} (2000–2014)	NCEP-DOE	9.8	41.3	51.1	20.8	60.2	81.0	0.8	2.3	3.1	1.4	9.5	10.9
	MERRA-Land	41.6	43.1	84.7	15.9	48.6	64.5	3.0	2.6	5.7	1.2	10.1	11.3
	MERRA-2	40.2	47.6	87.9	21.0	53.9	74.9	3.0	3.0	5.9	1.2	18.5	19.7
	ERA5	15.3	56.3	71.6	7.2	52.4	59.6	2.6	2.7	5.3	0.0	4.5	4.5
	ERA5-Land	18.2	56.3	74.4	8.8	56.2	65.0	2.9	2.5	5.4	0.0	6.4	6.4
	GLDAS-Noah v2.0	1.2	28.2	29.4	0.0	20.2	20.2	1.9	2.2	4.1	0.2	8.7	8.9
	GLDAS-Noah v2.1	18.7	47.7	66.5	14.7	46.0	60.8	4.2	4.5	8.7	0.0	6.8	6.8

Supplementary Figure 1. Spatial patterns of mean annual, mean annual maximum, and long-term maximum wetland extent from GIEMS-2, WAD2M, RFW, and G2017 respectively. The 0.25° × 0.25° grids with a <1% wetland fraction from RFW are masked out for all maps. Hatching in north of 40°N in Fig. (h) indicates there's no coverage for G2017.

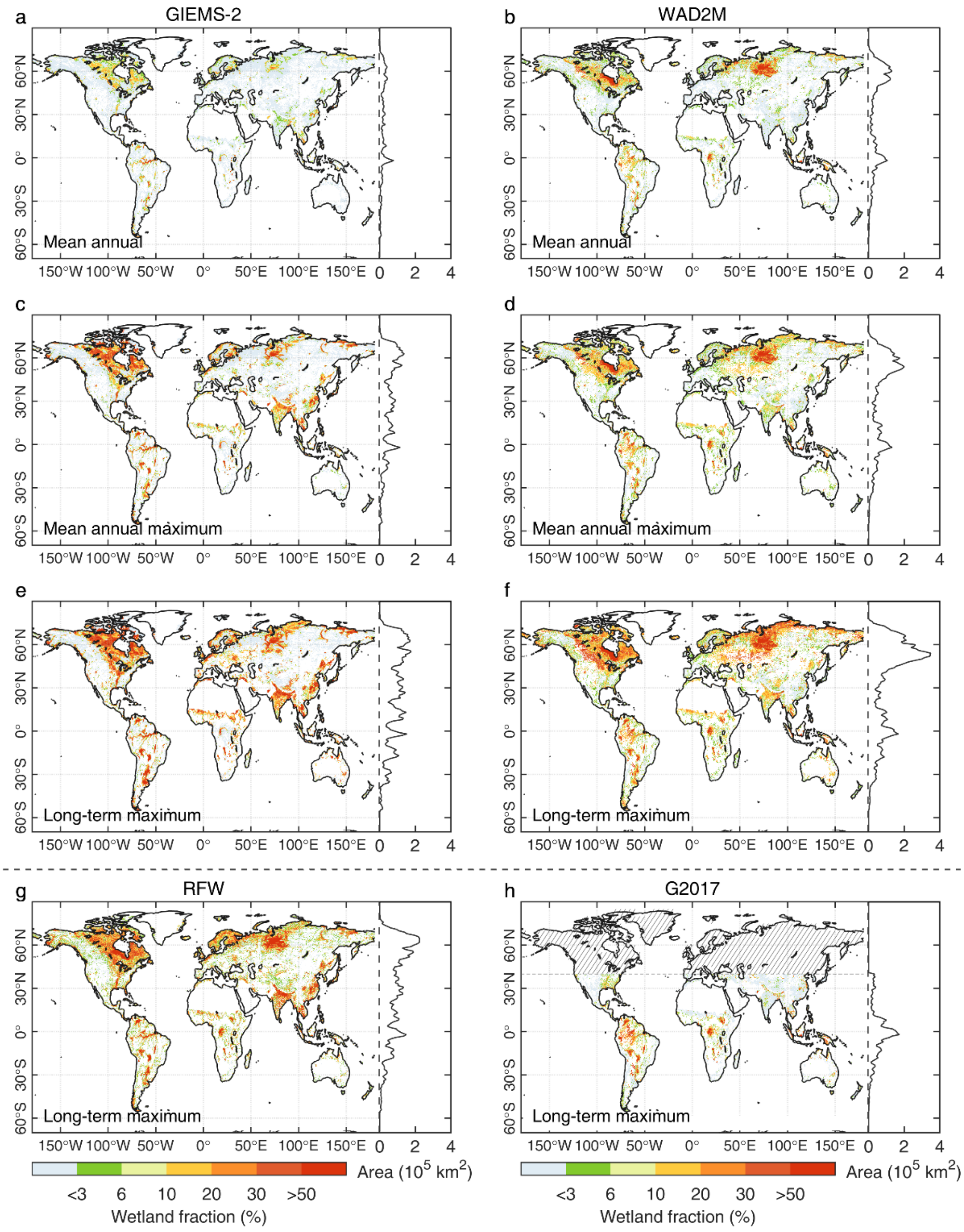

Supplementary Figure 2. Same as Fig. 3, but for simulations calibrated with RFW and G2017 (denoted as S_{RFW} and S_{G2017}). Hatching in north of 40°N in the right panel indicates there's no coverage for S_{G2017}.

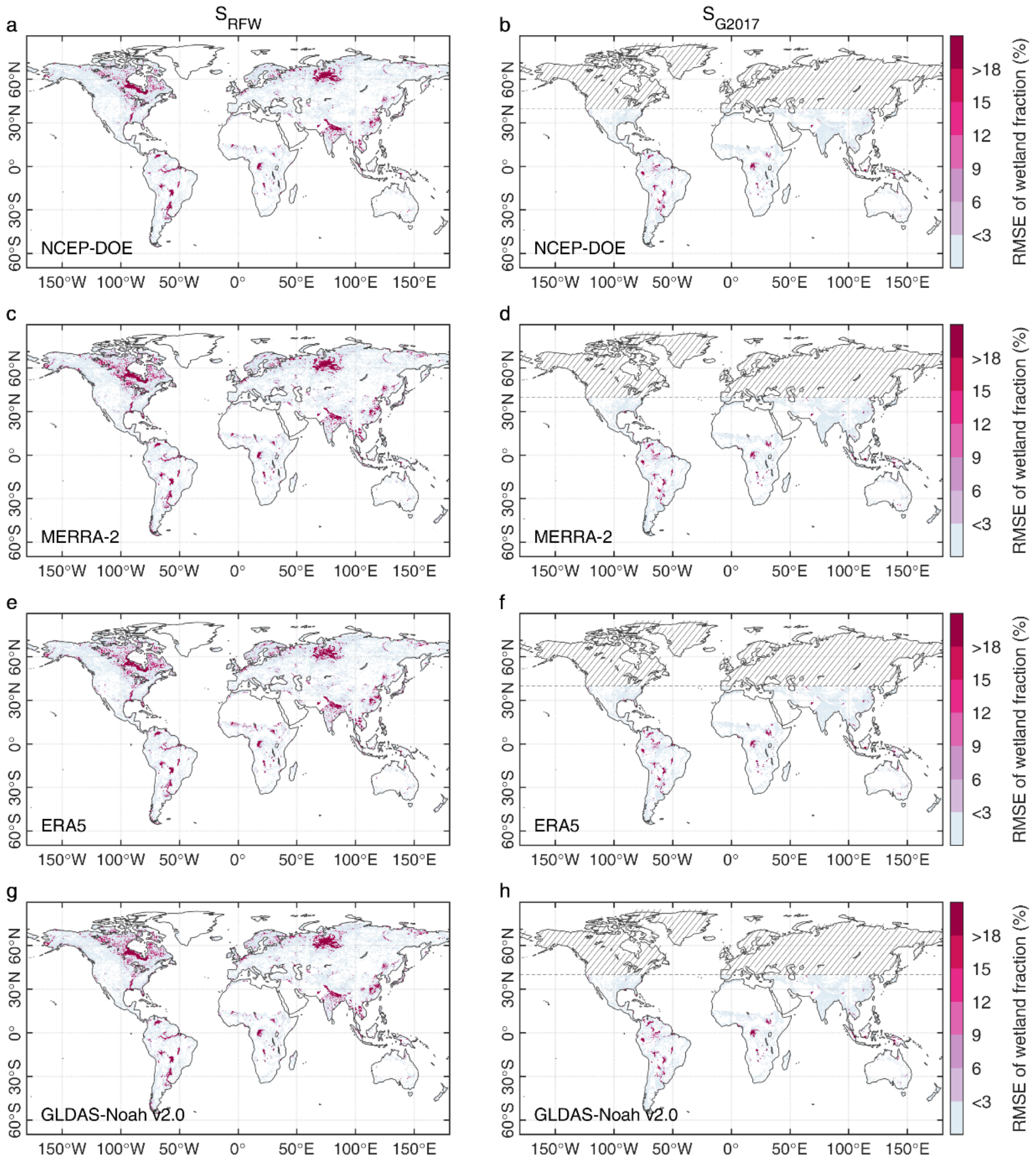

Supplementary Figure 3. Spatial patterns of simulated wetland extent. Simulated long-term maximum wetland extent based on four soil moisture data including NCEP-DOE, MERRA-2, ERA5, and GLDAS-Noah v2.0, with the parameters calibrated with GIEMS-2 and WAD2M (denoted as $S_{GIEMS-2}$ and S_{WAD2M}) respectively. The 0.25° × 0.25° grids with a <1% wetland fraction from RFW are masked out for all maps.

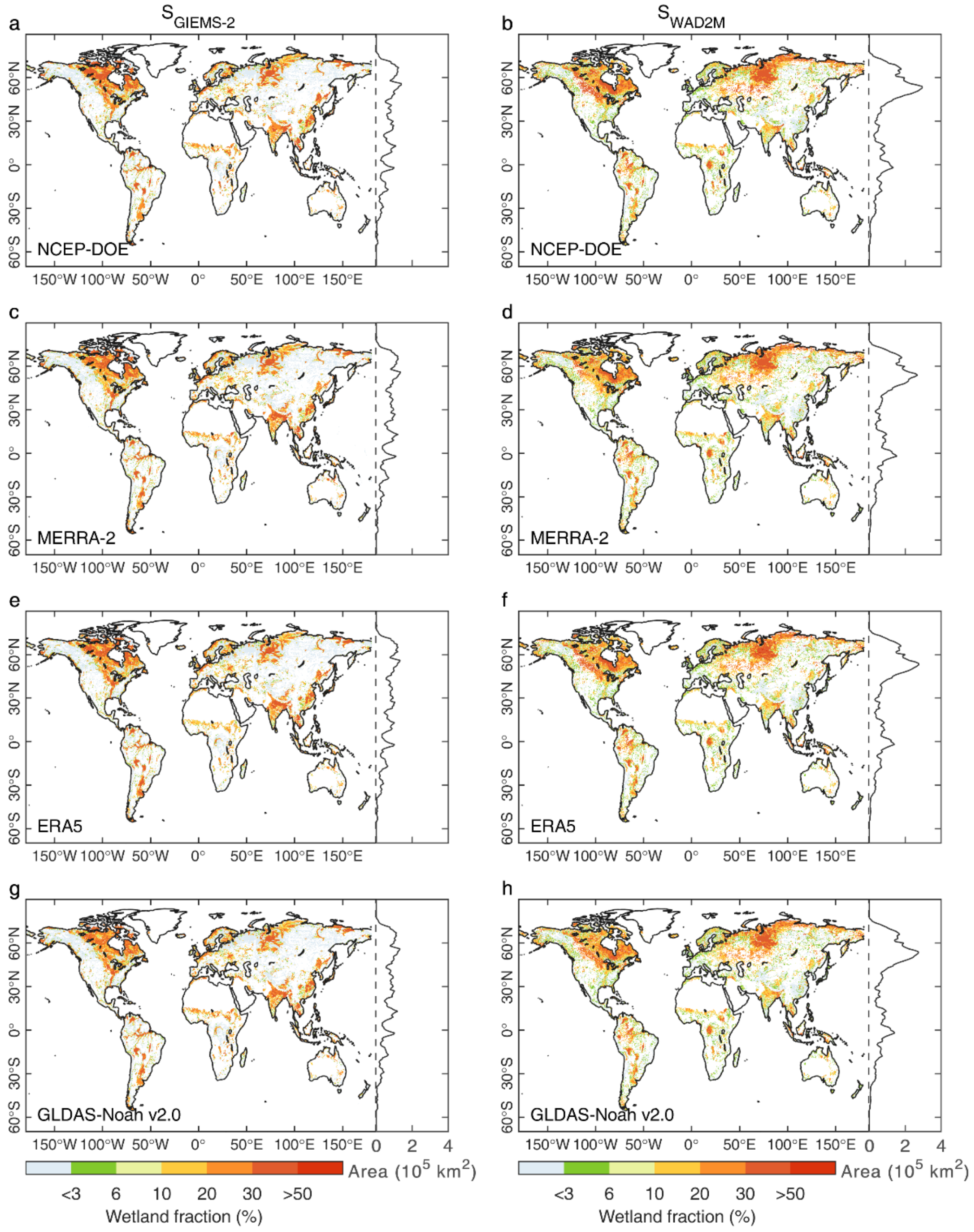

Supplementary Figure 4. Same as Supplementary Fig. 3, but for simulations calibrated with RFW and G2017 (denoted as S_{RFW} and S_{G2017}). Hatching in north of 40°N in the right panel indicates there's no coverage for S_{G2017}.

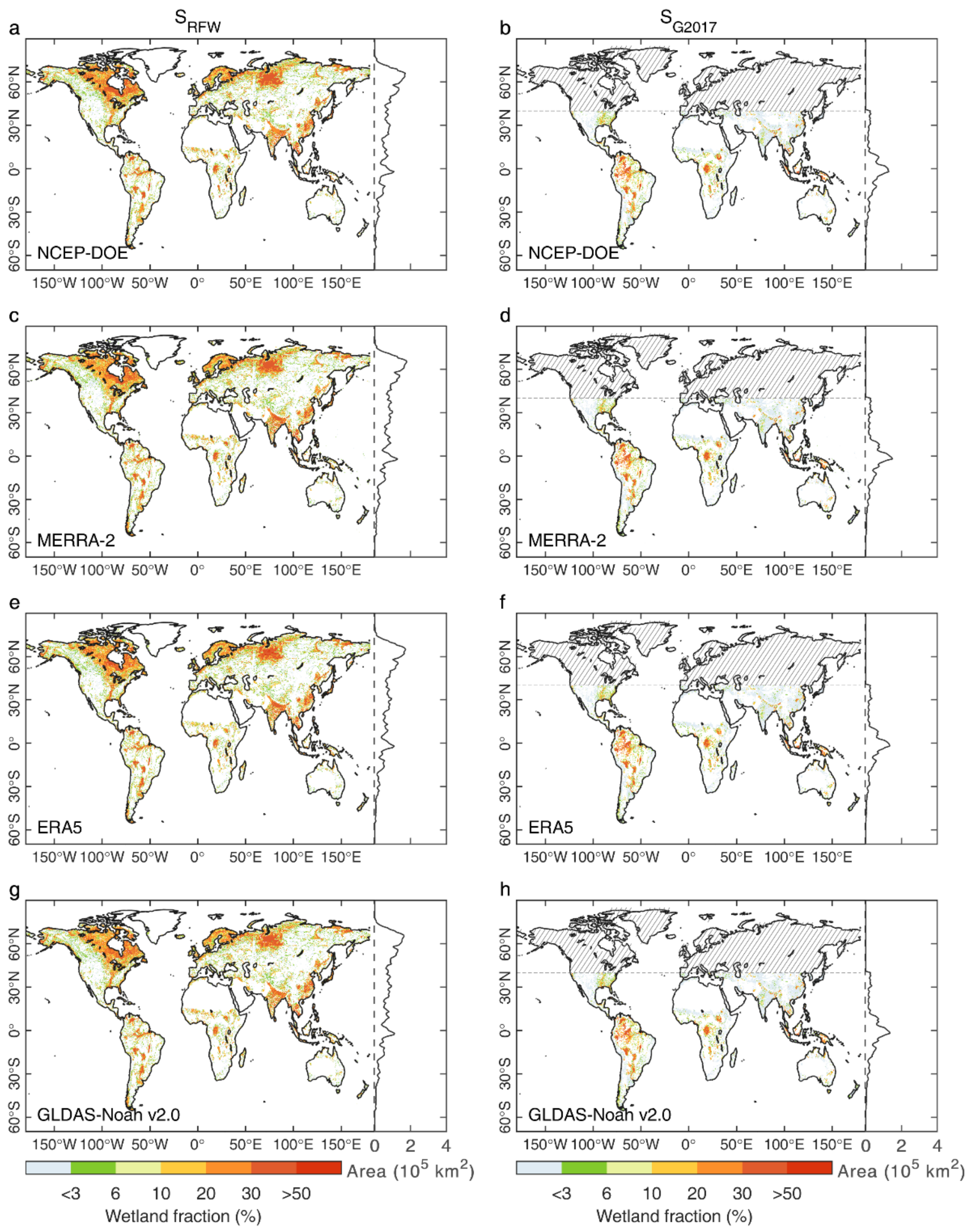

Supplementary Figure 5. Spatial distribution of the standard deviation (SD) of long-term maximum wetland fraction between (a) observed wetland data, (b) all simulations, (c) simulations based on the same soil moisture (SM) data (MERRA-2), as well as (d) simulations calibrated with same wetland data (RFW).

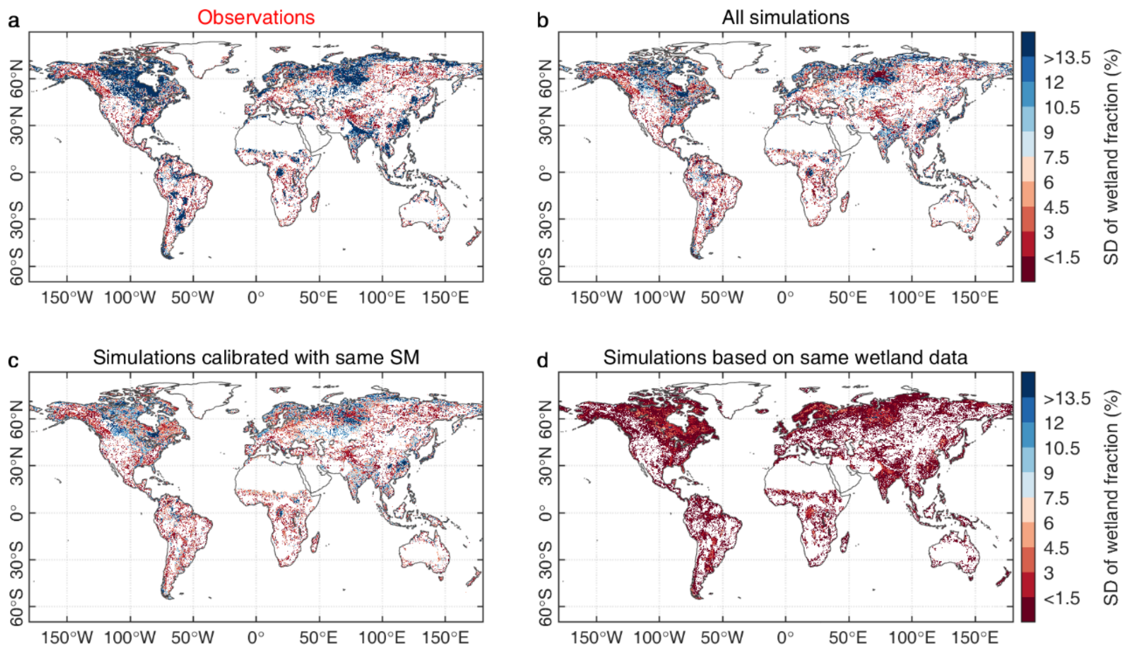

Supplementary Figure 6. The optimized parameter for simulations based on four soil moisture data including NCEP-DOE, MERRA-2, ERA5, and GLDAS-Noah v2.0, with the parameters calibrated with GIEMS-2 and WAD2M (denoted as $S_{GIEMS-2}$ and S_{WAD2M}) respectively. The 0.25° × 0.25° grids with a <1% wetland fraction from RFW are masked out for all maps.

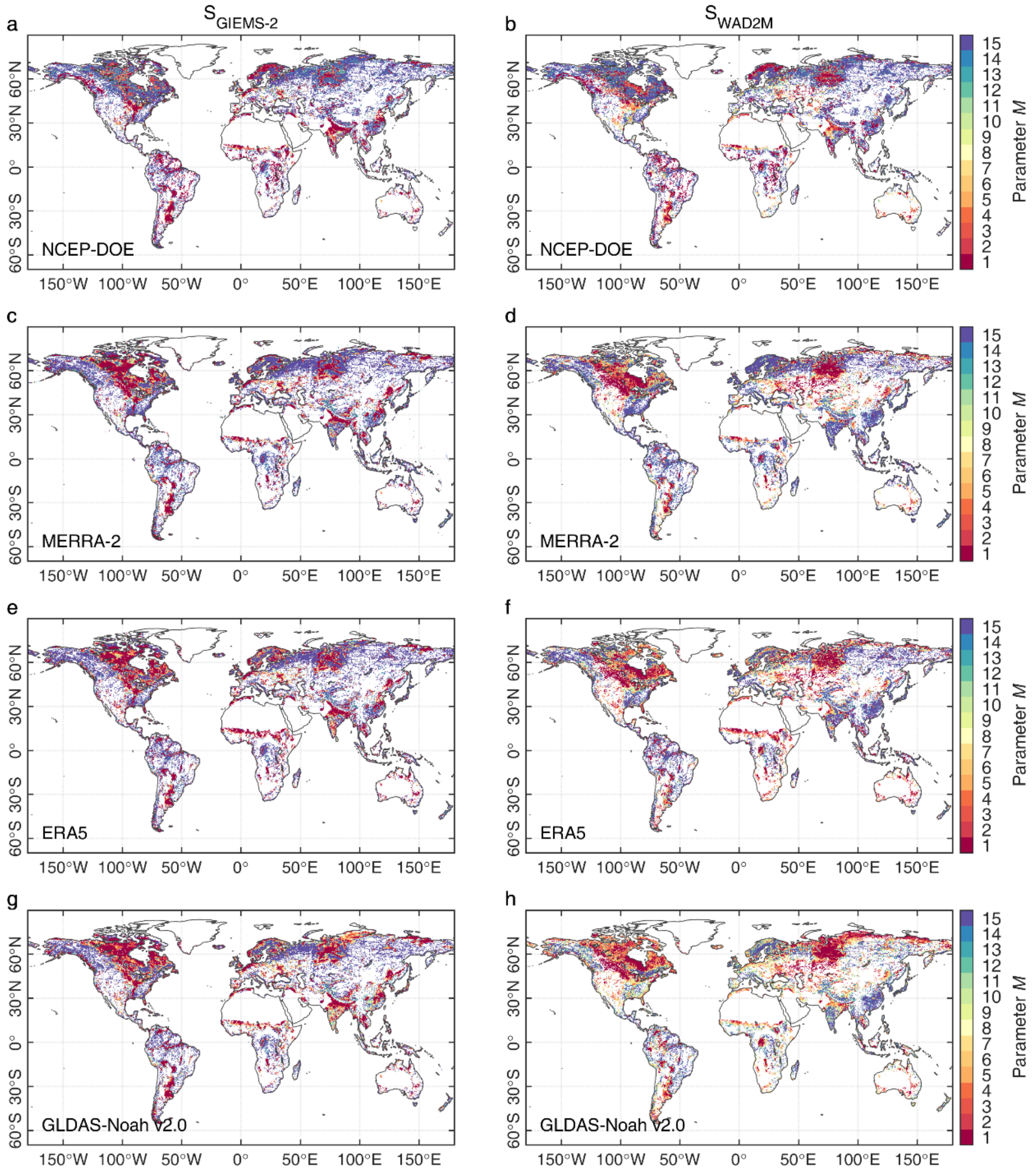

Supplementary Figure 7. Same as Supplementary Fig. 6, but for simulations calibrated with RFW and G2017 (denoted as S_{RFW} and S_{G2017}). Hatching in north of 40°N in the right panel indicates there's no coverage for S_{G2017}.

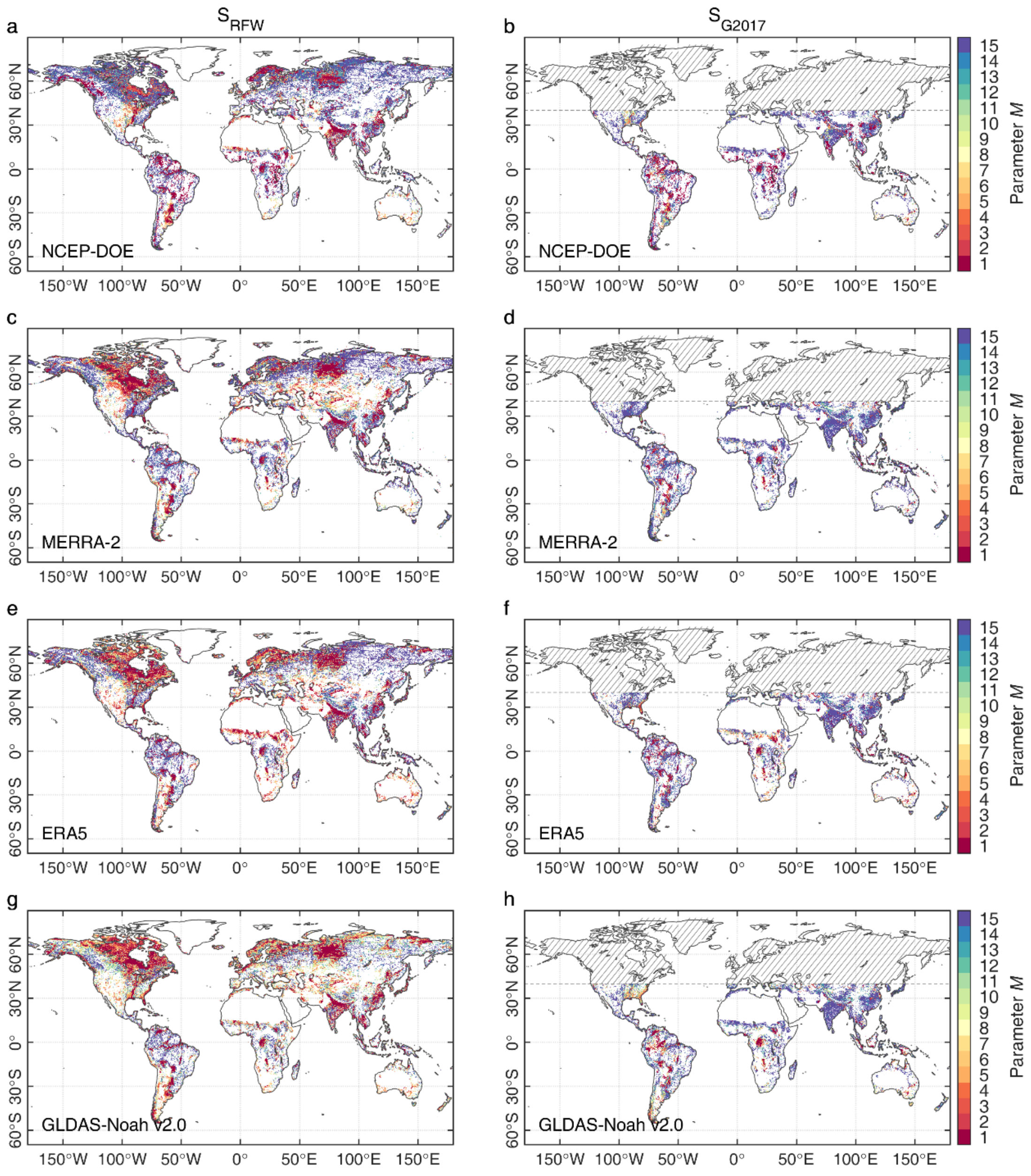

Supplementary Figure 8. Same as Fig. 6, but the parameters are calibrated with all months (Allm), mean seasonal cycle (Meanm), yearly maximum (YrMAX), and long-term maximum (MAX) wetland extent from GIEMS-2.

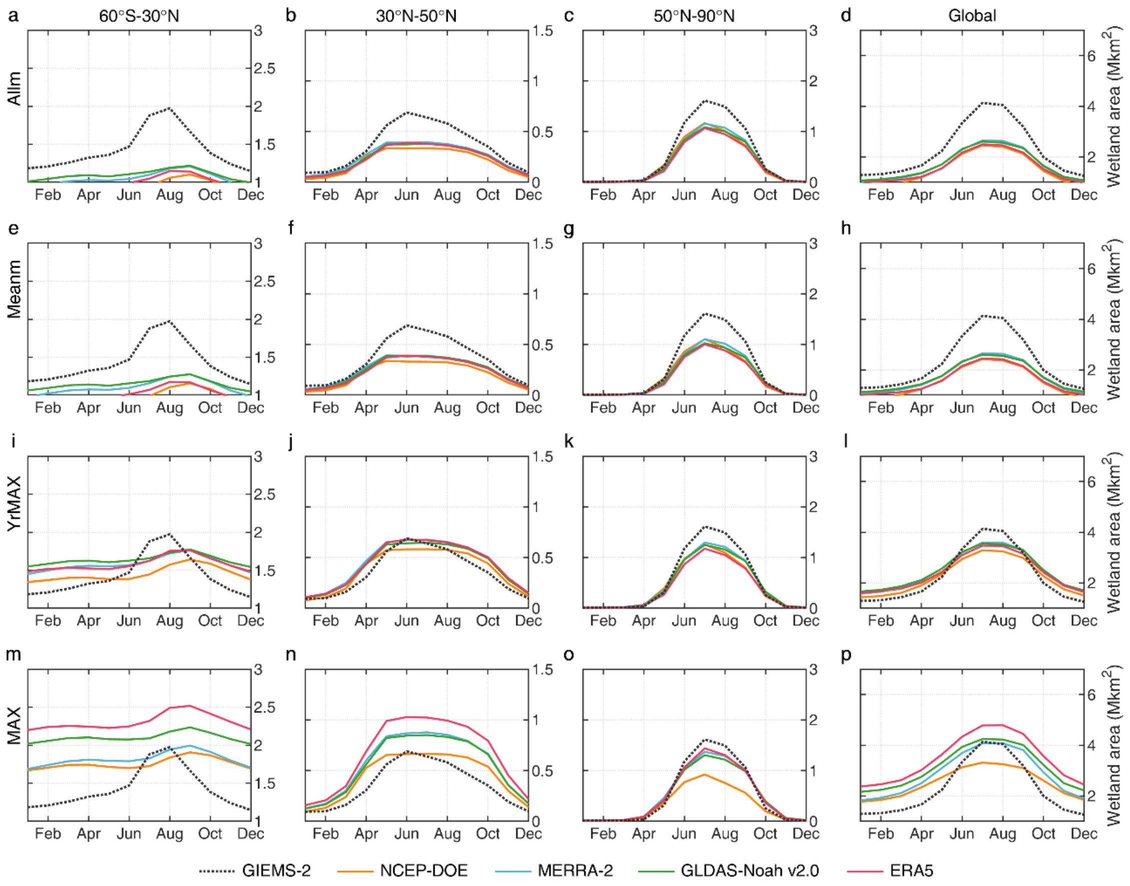

Supplementary Figure 9. Same as Fig. 6, but the parameters are calibrated with all months (Allm), mean seasonal cycle (Meanm), yearly maximum (YrMAX), and long-term maximum (MAX) wetland extent from WAD2M.

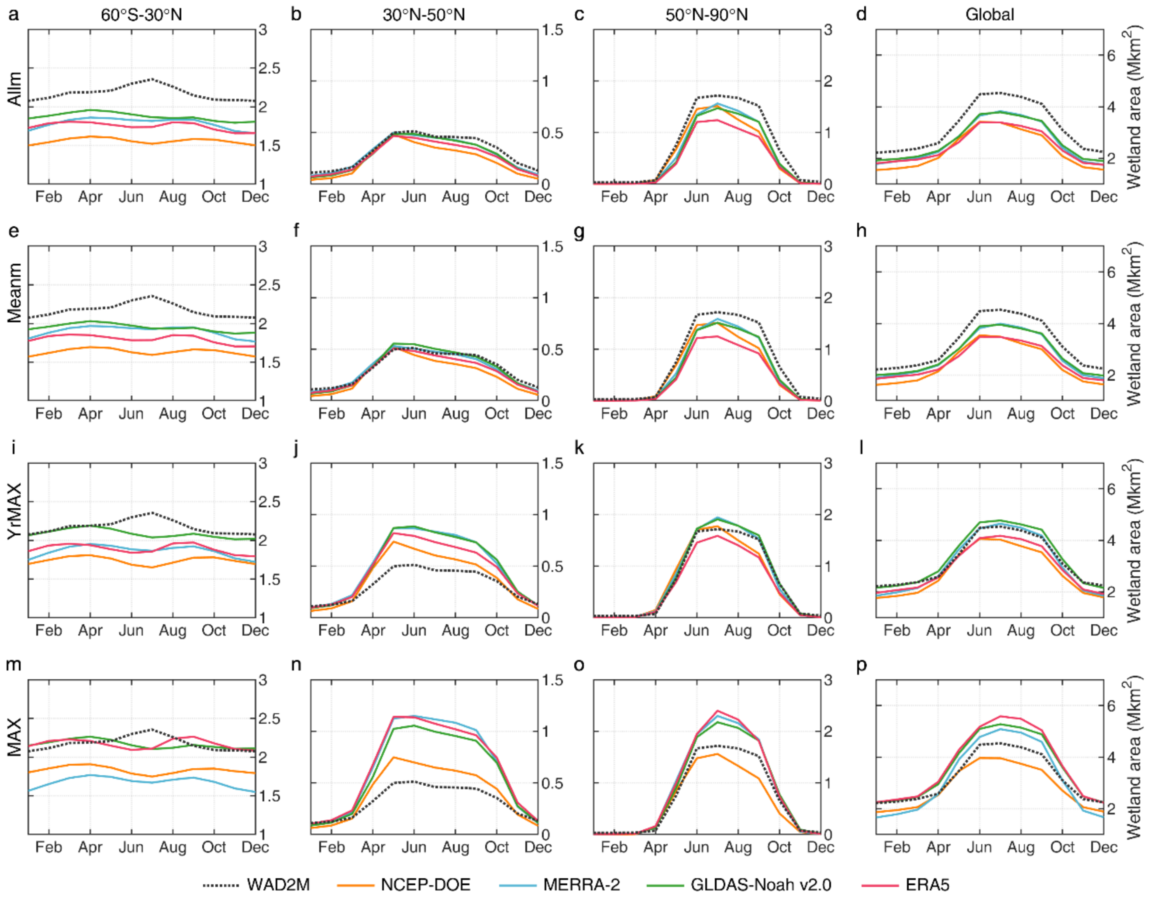

Supplementary Figure 10. Spatial distributions of the most probable month of maximum wetland extent from two observation data and simulations based on four soil moisture data including NCEP-DOE, MERRA-2, ERA5, and GLDAS-Noah v2.0, with the parameters calibrated with GIEMS-2 and WAD2M (denoted as $S_{GIEMS\text{-}2}$ and S_{WAD2M}) respectively. The 0.25° × 0.25° grids with a <1% wetland fraction from RFW are masked out for all maps.

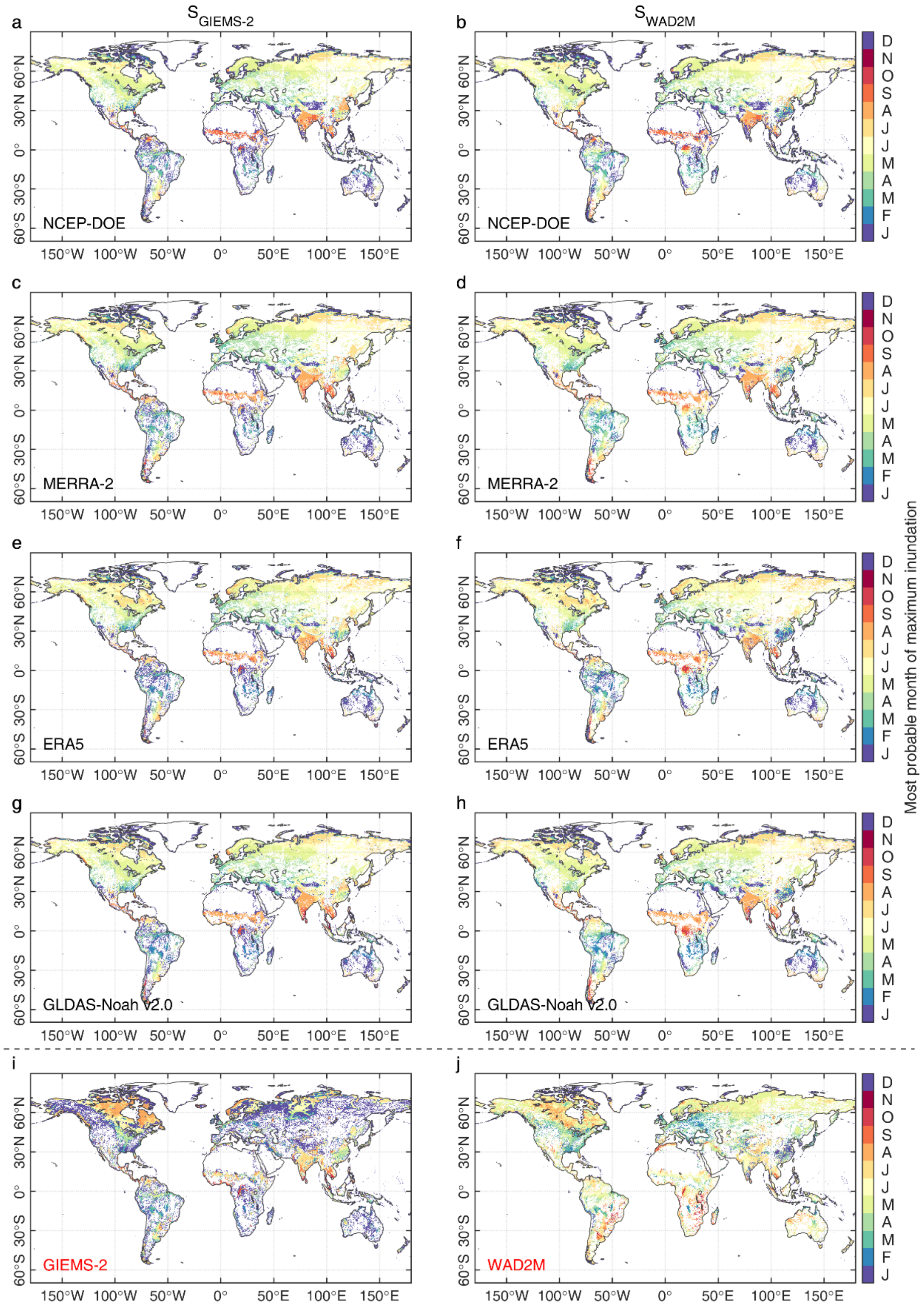

Supplementary Figure 11. Same as Supplementary Fig. 10, but for simulations calibrated with RFW and G2017 (denoted as S_{RFW} and S_{G2017}). Hatching in north of 40°N in the right panel indicates there's no coverage for S_{G2017}.

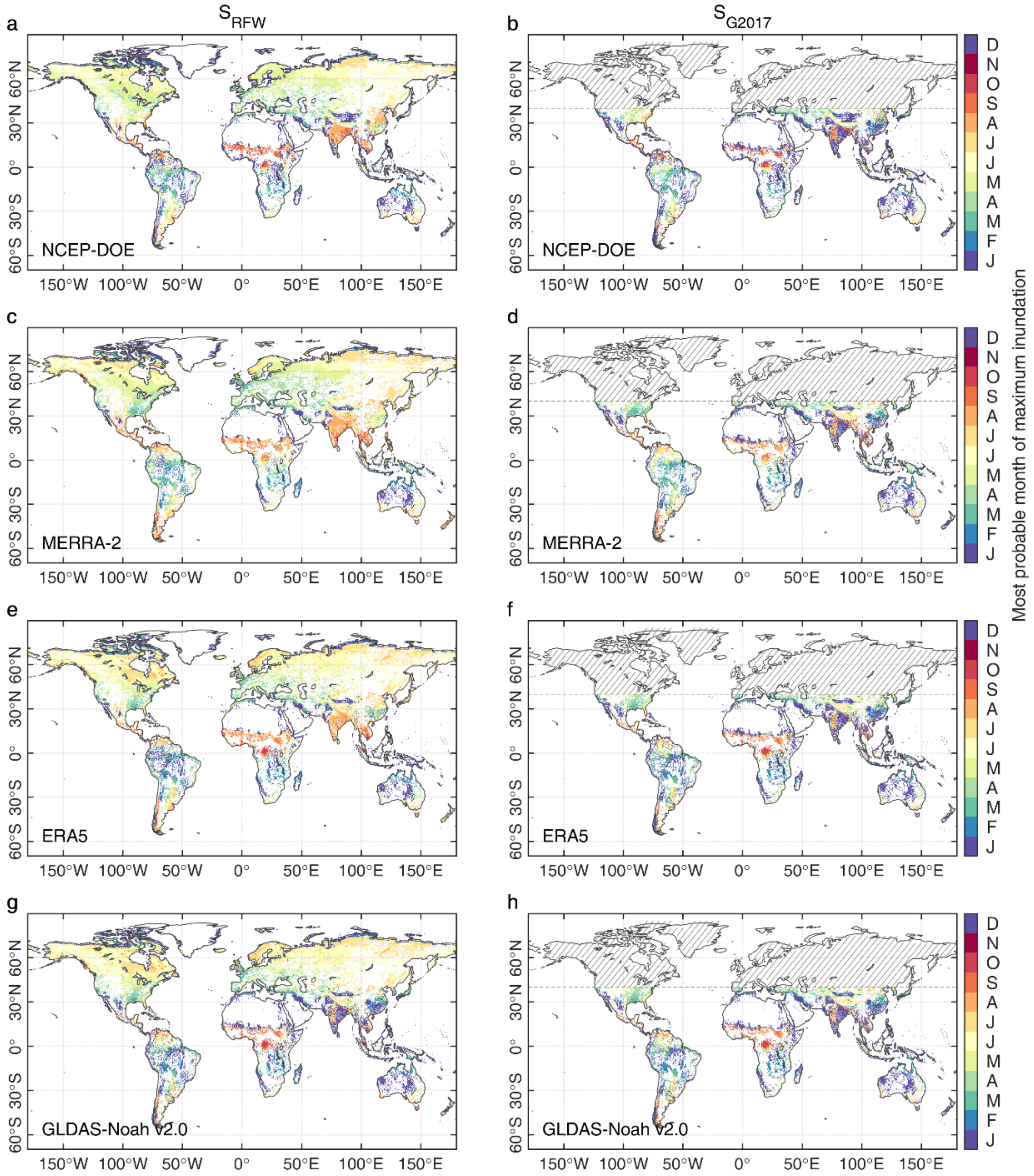

Supplementary Figure 12. Spatial distributions of standard deviation (SD) of wetland area anomaly from two observation data and simulations based on four soil moisture data including NCEP-DOE, MERRA-2, ERA5, and GLDAS-Noah v2.0, with the parameters calibrated with GIEMS-2 and WAD2M (denoted as $S_{GIEMS-2}$ and S_{WAD2M}) respectively. The 0.25° × 0.25° grids with a <1% wetland fraction from RFW are masked out for all maps.

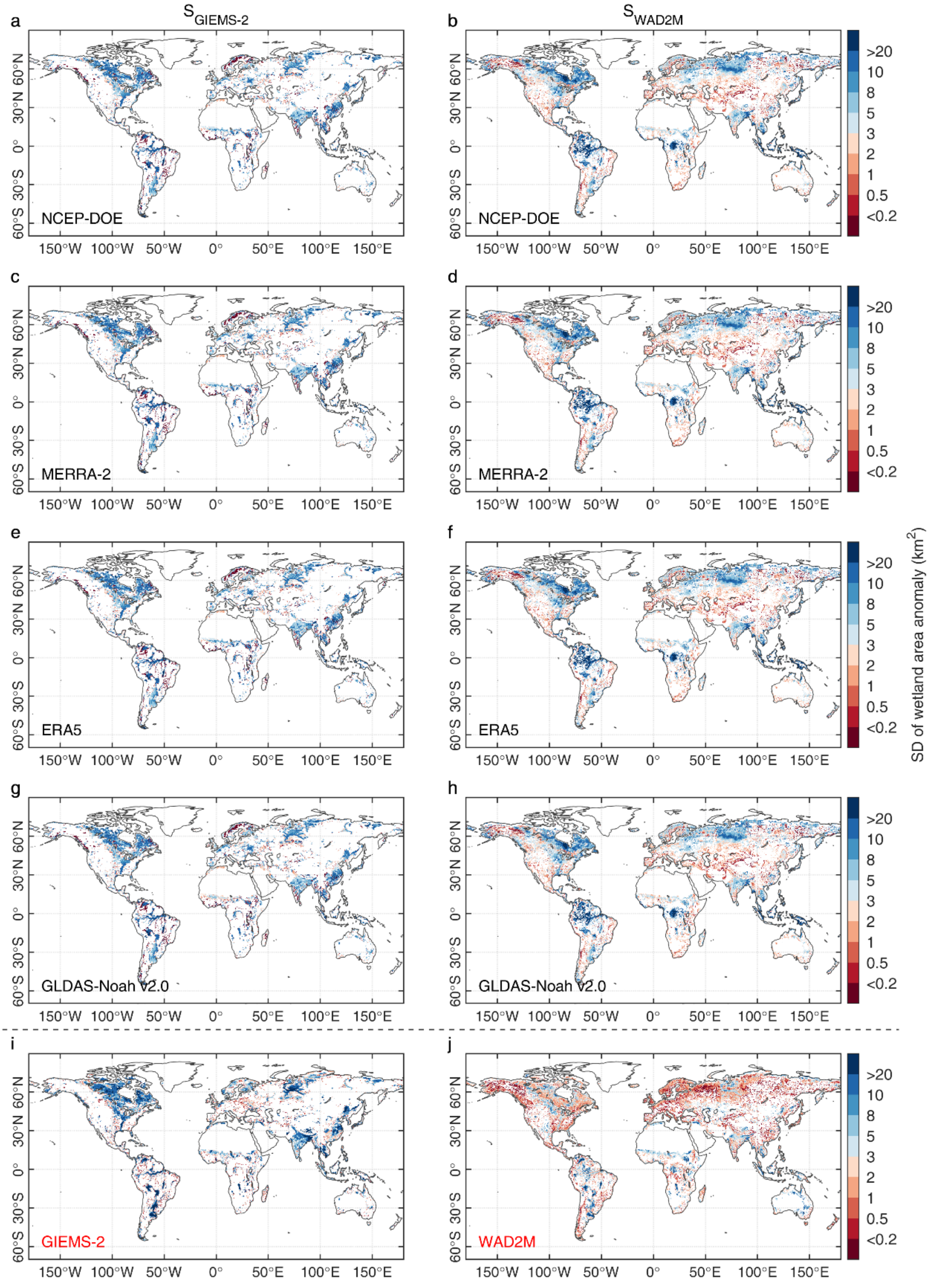

Supplementary Figure 13. Same as Supplementary Fig. 12, but for simulations calibrated with RFW and G2017 (denoted as S_{RFW} and S_{G2017}). Hatching in north of 40°N in the right panel indicates there's no coverage for S_{G2017}.

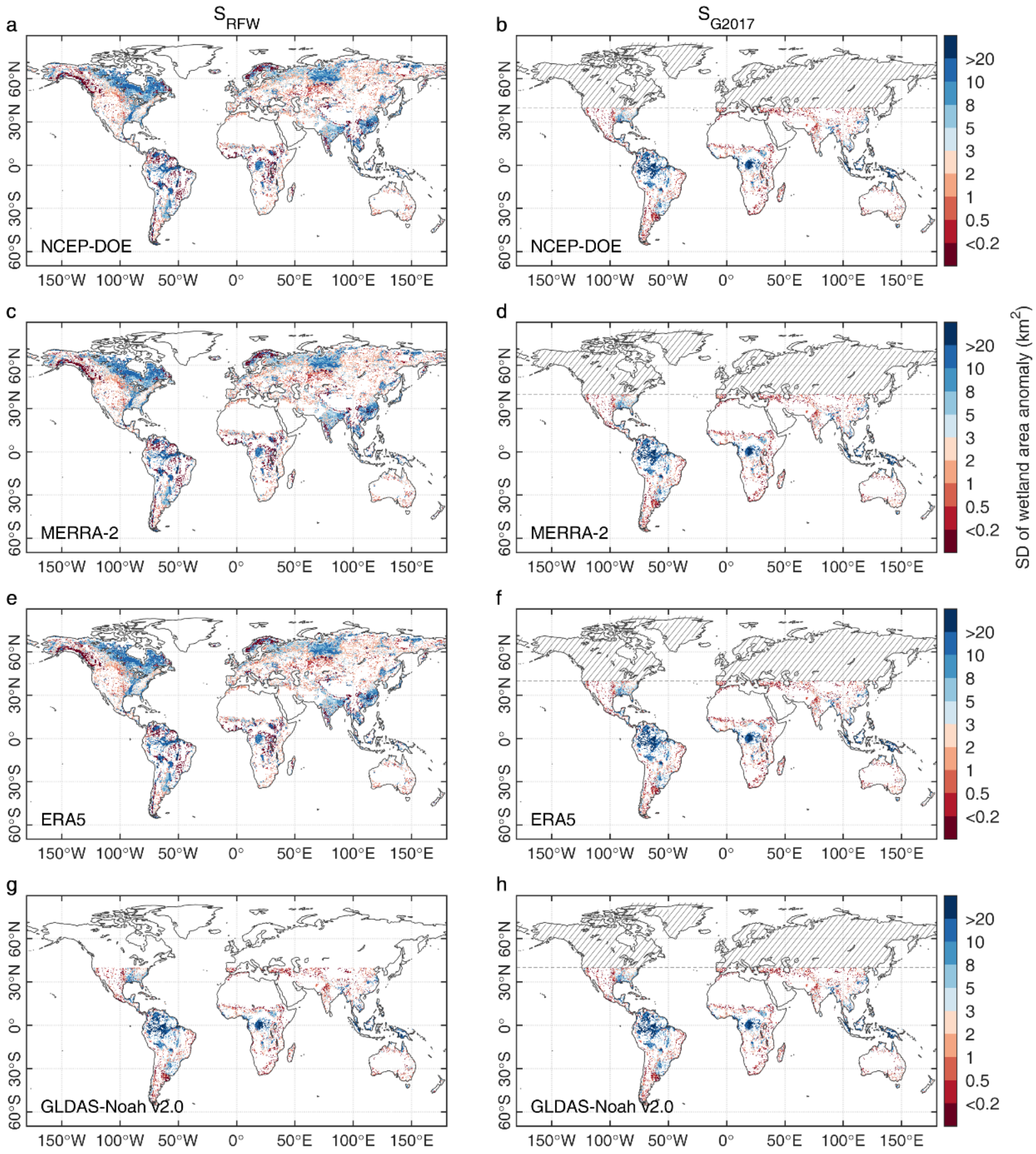

Supplementary Figure 14. Same as Fig. 9, but for simulations calibrated with RFW and G2017 (denoted as S_{RFW} and S_{G2017}). Hatching in north of 40°N in the right panel indicates there's no coverage for S_{G2017}.

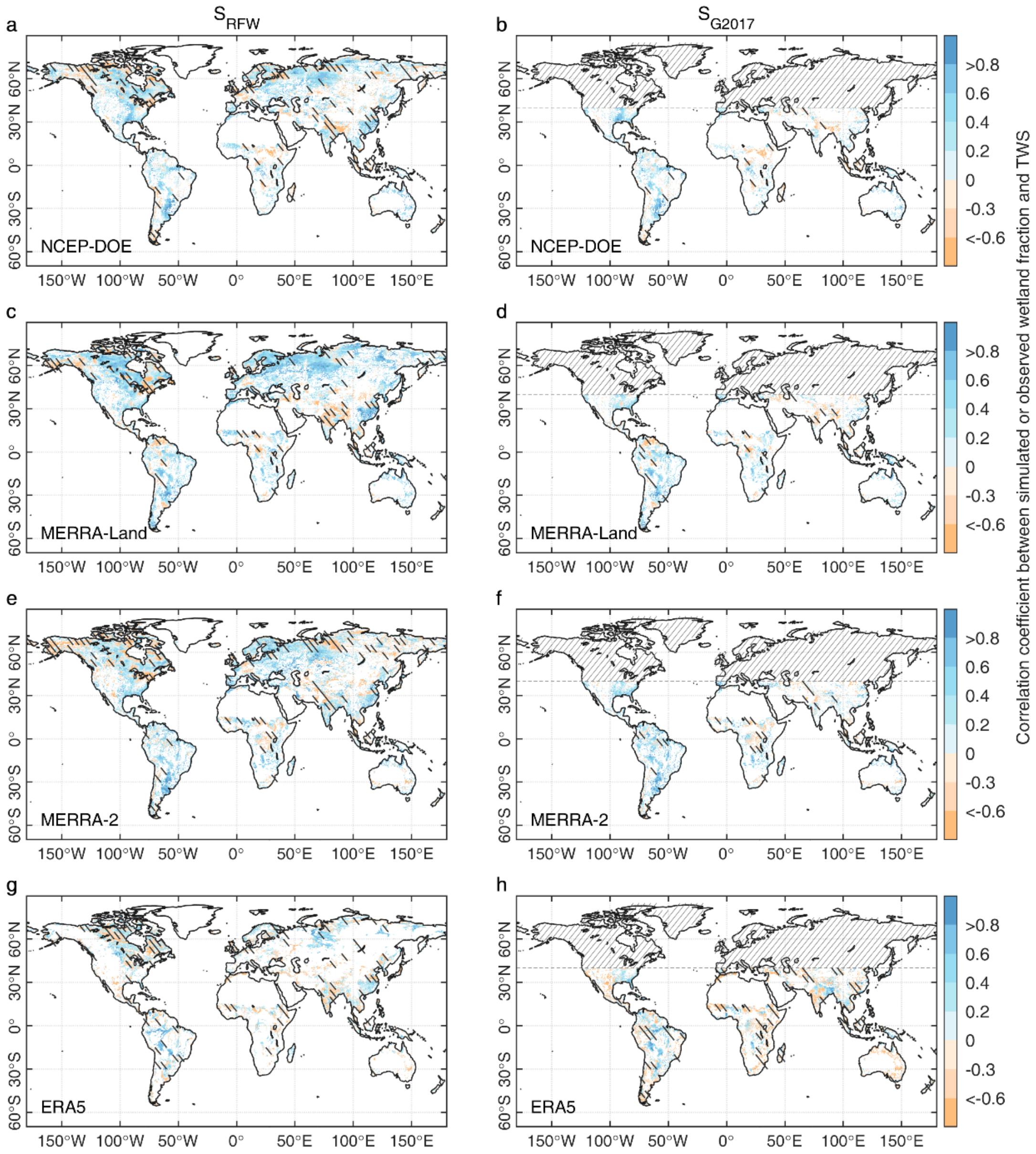

Supplementary Figure 15. Spatial pattern of the comparison of long-term maximum wetland extent between RFW and the optimized simulation only with tunable M based on soil moisture from MERRA-2 (a) and three grids for each case (b–d). The three cases indicate 1) simulated MAX wetland fraction is always smaller than MAX wetland extent from RFW; 2) simulated maximum MAX wetland fraction when $M = 1$ is larger while simulated minimum MAX wetland fraction when $M = 15$ is smaller than MAX wetland extent from RFW; 3) simulated MAX wetland fraction is always larger than MAX wetland extent from RFW.

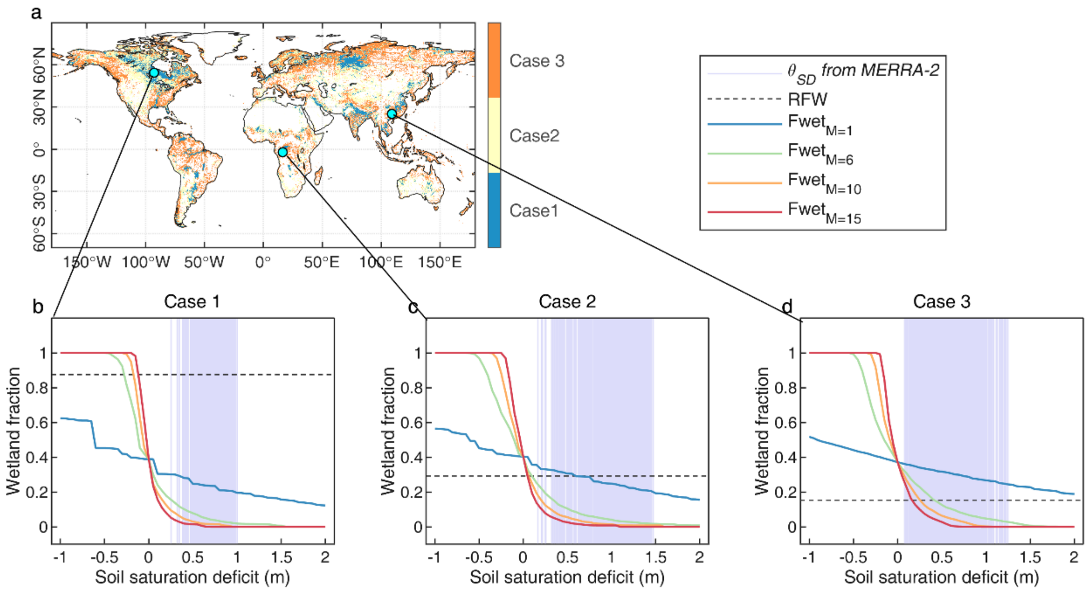

Supplementary Figure 16. Spatial distributions of mean soil moisture (SM) across all soil layers from seven reanalysis SM data sets. The periods of the seven SM data (NCEP-DOE, MERRA-Land, MERRA-2, ERA5, ERA5-Land, GLDAS-Noahv2.0, and GLDAS-Noahv2.1) cover 1980–2020, 1980–2015, 1980–2020, 1980–2020, 1981–2020, 1980–2014, and 2000–2020, respectively. The 0.25° × 0.25° grids with a <1% wetland fraction from RFW are masked out for all maps.

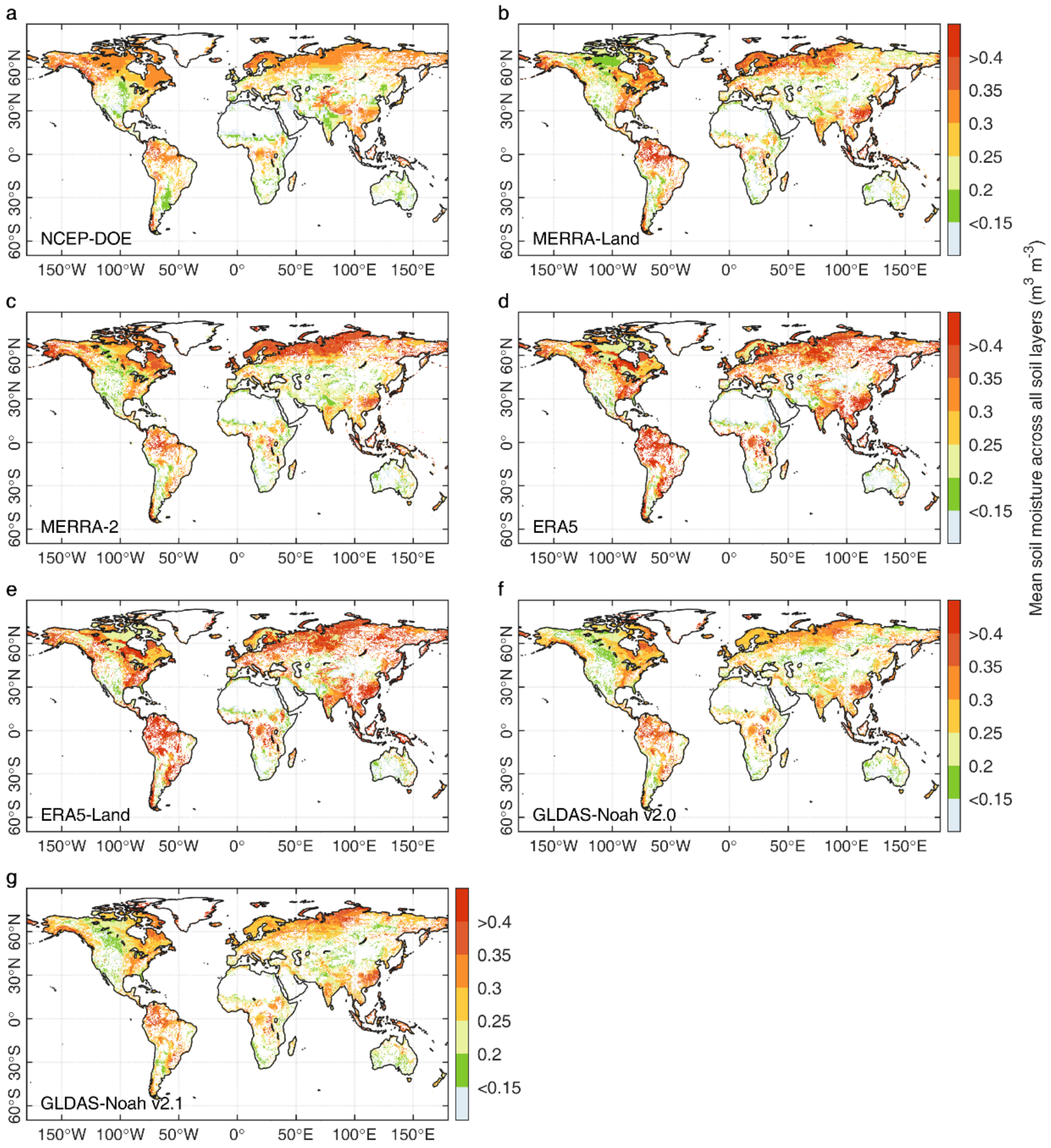

Supplementary Figure 17. Spatial distributions of correlations between mean soil moisture (SM) across all soil layers from seven reanalysis SM data sets and the terrestrial water storage (TWS) from GRACE for the period 2003–2016. The 0.25° × 0.25° grids with a <1% wetland fraction from RFW are masked out for all maps, and hatching indicates the correlations are statistically significant ($p < 0.05$).

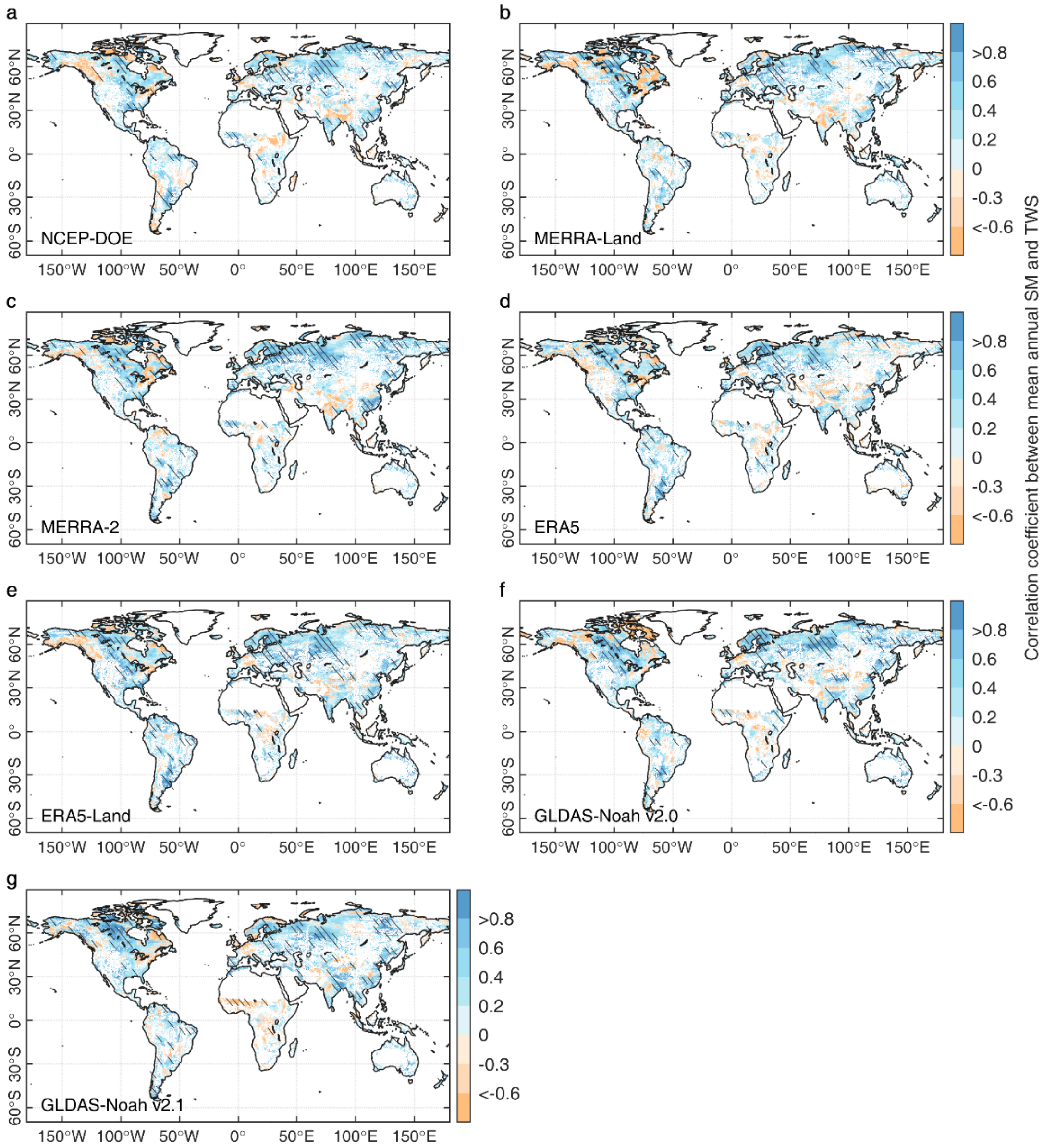

Supplementary Figure 18. Same as Fig. 7, but the parameters are calibrated with all months (Allm), mean seasonal cycle (Meanm), yearly maximum (YrMAX), and long-term maximum (MAX) wetland extent from GIEMS-2.

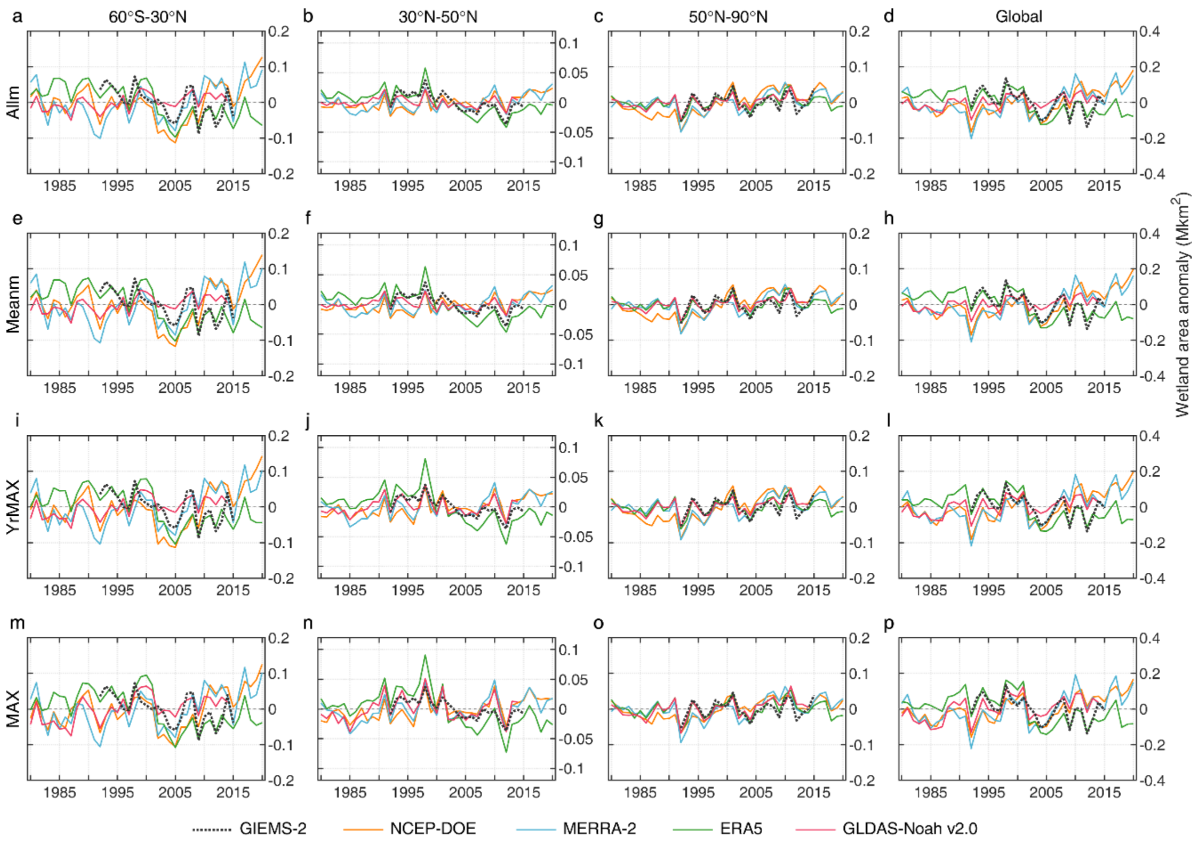

Supplementary Figure 19. Same as Fig. 7, but the parameters are calibrated with all months (Allm), mean seasonal cycle (Meanm), yearly maximum (YrMAX), and long-term maximum (MAX) wetland extent from WAD2M.

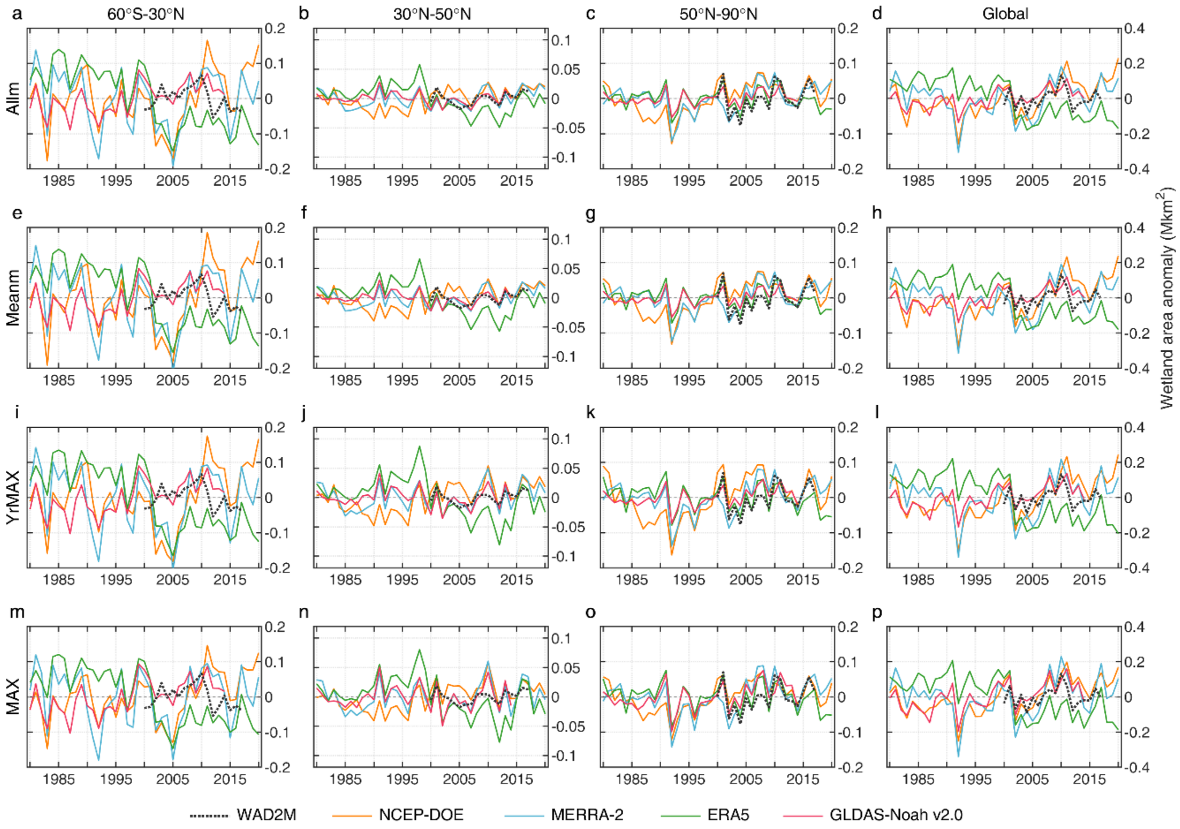

Supplement: Supplementary file 1 — Supplementary Information [file 41597_2022_1460_MOESM1_ESM.pdf]
